# Patient-centered modeling of the breast biopsy experience

**DOI:** 10.3389/frai.2025.1618357

**Published:** 2025-10-14

**Authors:** Isabel Nieto-Alvarez, Erik Bojorges-Valdez, Elvira Lang, Mohammadreza Ranaei Sharif, Göran Köber, Nicolas Rohleder, Oliver Amft

**Affiliations:** ^1^Chair of Digital Health, Friedrich-Alexander Universität Erlangen-Nürnberg, Erlangen, Germany; ^2^Intelligent Embedded Systems Lab., University of Freiburg, Freiburg, Germany; ^3^Siemens Healthcare AG, Erlangen, Germany; ^4^Department of Engineering Studies for Innovation, Universidad Iberoamericana, Mexico City, Mexico; ^5^Hypnalgesics, LLC, Brookline, MA, United States; ^6^Chair of Health Psychology, Friedrich-Alexander Universität Erlangen-Nürnberg, Erlangen, Germany; ^7^Hahn-Schickard-Gesellschaft für angewandte Forschung e.V., Freiburg, Germany

**Keywords:** patient experience, digital twin medical procedure, breast biopsy, NLP, linear mixed model (LME)

## Abstract

**Introduction:**

Despite significant advances in breast cancer screening and early detection over recent decades, rising patient volumes, limited resources, and time constraints hinder healthcare teams from anticipating distress and effectively managing the patient experience. We leveraged real-world data from 236 patients during a breast biopsy procedure and follow-up period.

**Objective:**

The study goal was to model important components of the multifaceted biopsy procedure and its effect on patient experience.

**Methods:**

We integrated data from patient-reported outcomes, psycho-social assessments, and workflow annotations.

**Results:**

We (1) provide a visual model of the patient pathway, (2) predict, with linear mixed models and machine learning, anxiety based on psychological pre-assessments as well as procedural events, and (3) analyze communication between caregiver and patient to understand moderators of the patient experience. Predictive modeling revealed significant correlation between psychological pre-assessments and median anxiety during biopsy (IES β = 0.91, CES-D β = 0.8, PSS β = 0.62, STAI β = 0.58, all with *p* < 0.001). Higher baseline stress was strongly associated with greater anxiety during biopsy. Centering each individual's procedure time at her first local anesthesia (LA) revealed a significant ($\beta_{t^{2}}$ *p* = 5.43*e*^−06^) temporal pattern in anxiety, which increased until LA and decreased afterwards. Using natural language processing, we identified patient expressions of pain and distress alongside workflow annotations.

**Conclusion:**

Our findings highlight the potential of combining data to model patient experience during a medical procedure. Our work helps to develop digital twins of medical procedures to support clinicians to provide proactive care and mitigate patient distress.

## 1 Introduction

The process of breast biopsy is stressful for women (Maimone et al., 2020; Grimm et al., 2024; Soo et al., 2019), involving various steps, such as preparation, the procedure itself, and subsequently waiting for results. However, patient experiences during each step remain poorly understood, making it difficult to anticipate points of distress. A digital twin of the process could provide transparency, enabling clinicians to better anticipate and respond to patients' needs. Image-guided core needle breast biopsy (CNB) is a diagnostic interventional procedure that involves local anesthesia, an incision in the breast, and insertion of a biopsy device guided by ultrasound or mammography. Typically, sedation is not used for ultrasound-guided procedures, during which patients lie on their back. For mammography-guided procedures, where patients lie on their abdomen, sedation is contraindicated. The biopsy process and patient pathway are not completed with the end of the procedure itself. Subsequently, patients recover briefly at the clinic and continue to wait up to 5 days for lab analysis results. Currently, anticipating disruptions in the process and understanding patient experiences throughout the journey remains a significant challenge. In healthcare, digital process or service models offer a novel approach to analyze workflows (Pesapane et al., 2022; Karakra et al., 2019), thus providing a framework to effectively track patients' experiences and aiding to minimize unexpected events.

Since artificially elicited emotions, e.g. in a lab, cannot be generalized to the real-world environment (Can et al., 2023) and the psychophysiological responses to artificial stimuli do not represent those in real life (Dantzer, 2016), we focus on a real-world analysis to partition and analyze the psychological pathway modeling problem. The patient experience, which includes triggers for distress, anxiety and pain in each moment of the pathway, could be represented by integrating multimodal data sources: patient-reported, contextual, and procedural data. Research shows that given the individual resources, people can communicate distress triggers and potentially regulate their response through coping strategies (Baker et al., 2005; Can et al., 2023). Although physiological monitoring and cortisol data are used in research on physiological distress detection (Dantzer, 2016), relevant data is typically not collected during current clinical workflows. Moreover, contextual data are useful to understand patient anxiety and distress. Data from the procedure, e.g., difficulty to find the lesion, difficulty to perform biopsy, or being sent home to wait for surgical biopsy, could provide insight into the individual experience. To deal with the complexity of the data and variables related to individual experience, recent studies in other domains use a concept map to visualize patient experience and moderating factors in a medical procedure (Nieto Alvarez et al., 2024; Falsiroli Maistrello et al., 2022; Gualandi et al., 2019).

Real-world data from the clinical process of CNB used in the present investigation was collected in a clinical trial dataset by Lang et al. (2006). At the time, the researchers assessed effects of relaxation on anxiety and pain levels of outpatients undergoing CNB diagnosis while being randomly assigned to three intervention groups during the biopsy procedure. Furthermore, Lang et al. (2009), examined the effects of uncertainty after the biopsy procedure on salivary cortisol levels and found that uncertainty was associated with significantly higher biomarkers signaling distress. In the present work, we focus on experience, including patient reported anxiety and pain, because these aspects increase patient management effort during procedures, increase resource utilization, and increase healthcare costs (Ladapo et al., 2018).

Our objective was to develop models of the breast biopsy procedure and the experience of patients based on actual CNB data. Specifically, we investigate: (1) patient experience of the biopsy process and critical events, (2) individual variables influencing the patient experience, e.g., psychosocial predictors, and (3) situational moderators of the patient experience. Our methods integrate diverse data and may lead to future procedural digital twin models of patient experience. Our work contributes to early identification of distressing moments, as well as to improve the care experience along the patient pathway.

## 2 Results

We investigated the procedural and psychological pathway using a CNB study dataset (Lang et al., 2006, 2009) involving 236 patients during the biopsy procedure and a 5-day follow-up period at home.

### 2.1 Process model and moderating factors

**Figure 1:**
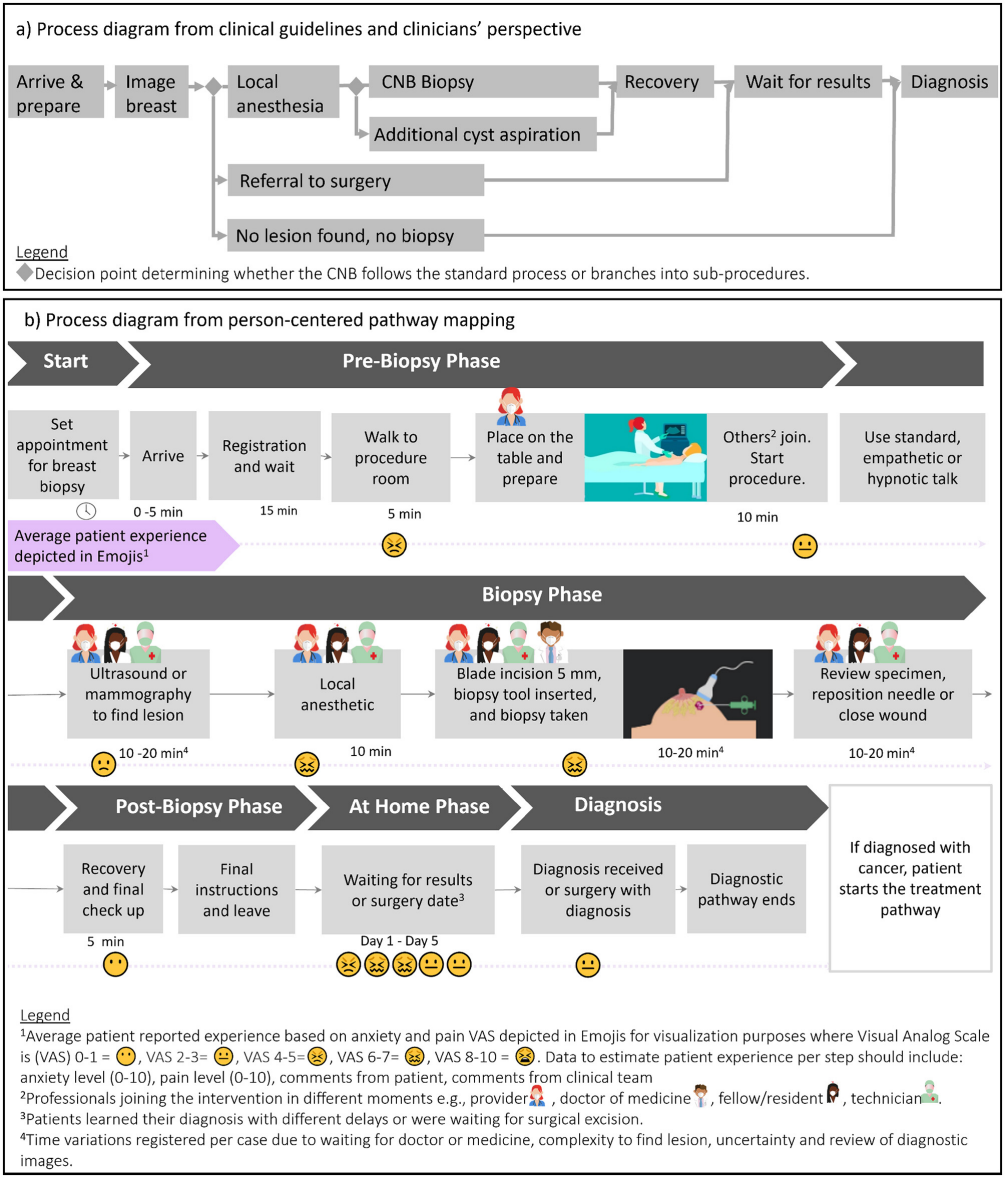
Process modeling for patients undergoing CNB. **(a)** Process diagram based on clinical guidelines and identified sub-procedures from real-world clinical perspective. Process diagram **(b)** of the person-centered pathway mapping, including patient's perspective, averaged experience report according to our dataset, healthcare professionals joining/leaving the procedural room, time for the process step duration or wait time.

We describe the biopsy process based on clinical guidelines and real-world sub-procedures, see Figure 1a. A detailed person-centered pathway (Figure 1b) maps the patient experience from scheduling appointment for CNB, through the procedure, and during the post-procedural period at home. The pathway diagram displays real-world process variations, which may impact experience of care, e.g., various healthcare professionals joining the intervention room, time for the process, and variation in delay before patients receive results. Biopsy results were received on day 5 by 16 patients (13%) with malignant findings and 37 patients (29%) with benign diagnoses. For 73 patients (58%), results were delayed: (a) the result had not been communicated yet (*n* = 54); (b) CNB could not be performed and patients needed to wait for surgical excision (*n* =14); (c) histopathologic analysis of the biopsy revealed at-risk lesions (*n* = 4) or benign cells (*n* = 1), recommending surgery for excision and follow-up diagnosis.

#### 2.1.1 Procedural sub-classification

Although all patients (*n* = 236) were scheduled for CNB, we implemented a semi-automated methodology to classify patients into sub-procedures, based on 4,411 textual observations. Sub-procedure categories were: (1) no biopsy after diagnostic imaging (*n* = 23), (2) standard imaging with local anesthetics and biopsy (*n* = 177), (3) require further procedure after imaging (e.g., surgery) (*n* = 32), (4) neither imaging nor biopsy (*n* = 1).

**Figure 2:**
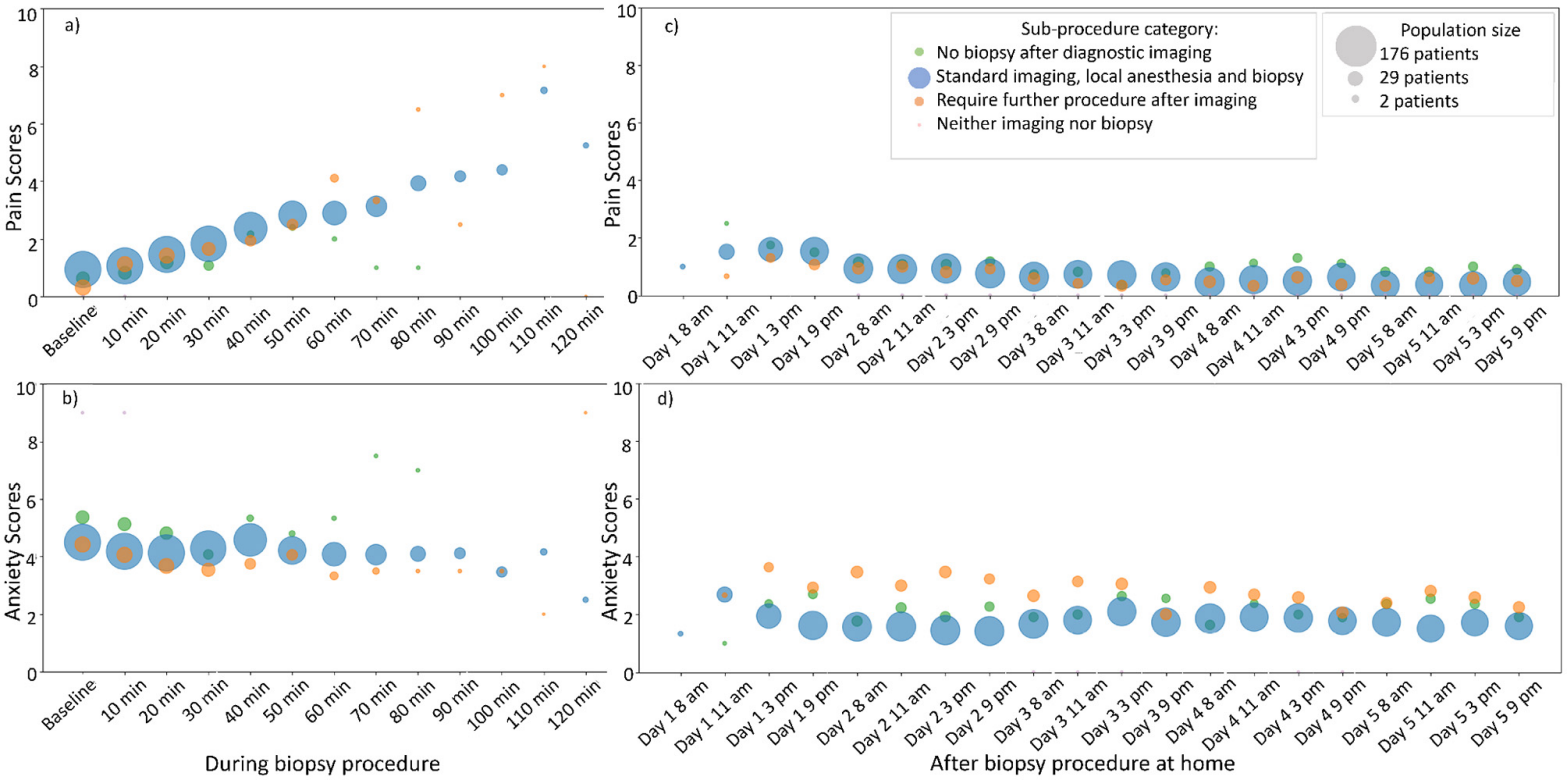
Patient reported experience during CNB procedure and five days post-procedure. **(a, c)** Patient reported pain. **(b, d)** Patient reported anxiety. During the CNB procedure, patients reported every 10 min. During post-procedure, patients reported at four moments during the day, over five days. Sub-procedures were identified from unstructured data analysis, see methods section for details.

Figure 2 shows patient reported scores during the procedure and for 5 days post-procedure. Data on anxiety and pain were available from arrival through 5 days post-procedure at home. Descriptive statistics revealed higher anxiety at the start of the procedure compared to its end. Post-procedure anxiety was elevated, if CNB procedure result included a request to perform another intervention (e.g., surgery), compared to cases were it was not.

Sub-classification of procedures represents intra-procedure information and cannot predict median anxiety during the procedure. Instead, we used sub-classification to predict “next minutes,” subsequent days, and dynamic models (e.g., dynamic prediction with landmark model). However, due to limited data the results lacked statistical power, preventing significant probabilistic associations with other variables.

**Figure 3:**
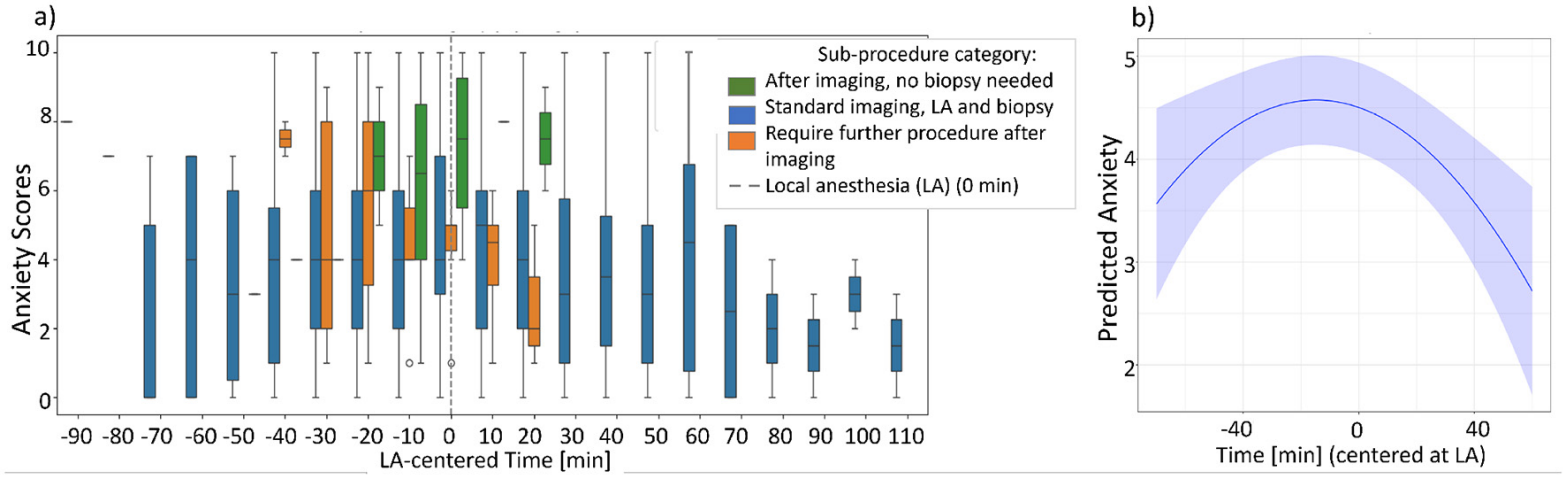
Predictability of local anesthesia (LA) as a first moment with higher anxiety. **(a)** LA-centered graph per sub-procedure and **(b)** the LA as a significant moment for anxiety, effect plot analyzed with linear mixed model.

We applied a semi-automated word recognition method to identify the first local anesthesia (LA) moment in unstructured data. Figure 3a shows an LA-centered graph grouping patients, who received anesthesia by sub-procedure. Using a linear mixed model, we tested the predictability of LA as peak anxiety moment. Centering time at LA revealed a significant (flipped U-shape effect plot β_*t*_ with *p* = 0.61 and $\beta_{t^{2}}$ with *p* = 5.43*e*^−06^) increase in anxiety shortly beforehand (see Figure 3b), underscoring its critical role in the procedure. Notably, centering time at the grand mean yielded insignificant parameter estimates.

#### 2.1.2 Labeling critical events in the process

Human expert analysis of the unstructured data further sub-labeled normal biopsy procedures by critical events, using terms from Table 1 to define each patient's process. Supplementary Figure S3 illustrates pain and anxiety levels experienced during these events. Sub-labels included: biopsies in two breast areas (*n* = 6), cyst aspiration (*n* = 8), clinician consultations (*n* = 4), patient-triggered events (e.g., restroom visit, *n* = 9), clinician-triggered events (e.g., fainting, *n* = 2), waiting for clinicians (*n* = 22), technical complications (e.g., imaging difficulties (*n* = 9), and technology malfunctions (*n* = 2). The sub-classification and sub-labeling of events capture process variations during CNB, and define classifiers based on real-world data to be used for potential future digital twin models of medical procedures.

**Table 1:** Sub-labeling based on words or observations from unstructured data to identify events happening per patient during CNB.

**Sub-labeling in the procedure**	**Related words from unstructured data**
Double area biopsied	2nd area, other breast, 2nd Bx, second bx
Consultation needed	Needs help, Dr [...] and Dr [...] enter, consult
Patient event	Hostile, restroom, moved, drunk, touched sterile space, request to scan left breast too
Clinician event	Faint (syncope), says study is disturbing factor
Waiting for clinician	Waiting, patient alone, waiting for, tech/MD out, tech/MD back
Technical complications	Complication(s), (re)positioning, [research] study as a disturbing factor
Technological complications	Machine very loud, machine off, problems with machine, computer down, cannot continue, problems with computer, needle doesn't open, needle needs replacement, new device, salesperson in room, patient uncomfortable with man in room

### 2.2 Factors influencing individual experience

**Figure 4:**
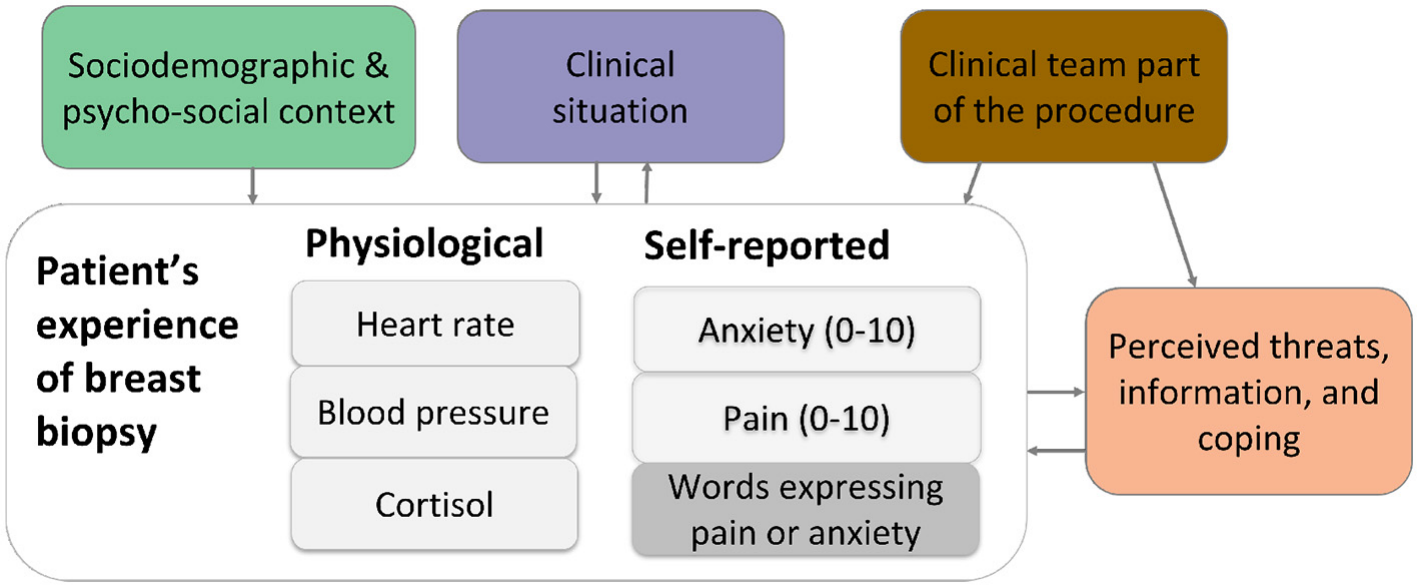
Concept map of patients' experience and moderating variables. The patient experience includes physiological and self-reported data, the concept map identifies the relation with moderating variables and is the basis for analysis of relations of variables.

The concept map in Figure 4 organizes variables moderating patient experience into three groups: (1) sociodemographic and psycho-social variables, (2) clinical factors, and (3) situational variables including clinical team involvement, perceived threats, and coping resources. While probabilistic relations were established for some psycho-social variables and situational variables (e.g., clinical team involvement, and coping mechanisms), limited data prevented statistical significance across all variables. Further details on the available data for each group are provided in the Methods section.

#### 2.2.1 Psychosocial predictors

Descriptive analysis (Supplementary Figure S1), supported by a linear mixed model, revealed significant correlations between median anxiety and pre-procedure psychological assessments. The assessments included the Impact of Event Scale (IES), Perceived Stress Scale (PSS), State-Trait Anxiety Inventory (STAI), and the Center for Epidemiological Studies-Depression Scale (CESD) from 120 patients. While most individuals reported lower to moderate levels of anxiety (most measures are right-skewed), a notable subset experienced high anxiety levels, suggesting disproportionate vulnerability in some patients. Linear mixed model results showed the strongest predictor was IES (β = 0.91), followed by CES-D (β = 0.82), PSS (β = 0.62), and STAI (β = 0.58), all with *p* < 0.001.

#### 2.2.2 Socio-demographic predictors

Multidimensional descriptive analysis revealed variability in anxiety levels across socio-demographic factors. Anxiety and pain scores showed no clear trend with age, suggesting its influence may be complex or nonlinear. Race and ethnicity exhibited some clustering by anxiety scores, potentially reflecting cultural or socio-environmental influences; however, under representation of certain racial groups limits broader conclusions.

#### 2.2.3 Physiological predictors

Baseline physiological measures (e.g., blood pressure, heart rate) from 101 patients, showed low correlations with median anxiety scores. Cortisol levels as an indicator of anxiety presented challenges for analysis. Although pre-post procedure measurements include less confounding factors than “at home” data, none yielded significant results in the linear mixed model. Potential confounder factors include: age variability (e.g., young and older patients), hours of sleep before the procedure, and hormonal differences (e.g., pregnancy, menopause, hormone replacement therapy) with progesterone and estradiol levels potentially influencing sensitivity to distressing situations.

### 2.3 Situational moderators of experience

#### 2.3.1 Clinical team's impact on experience

**Figure 5:**
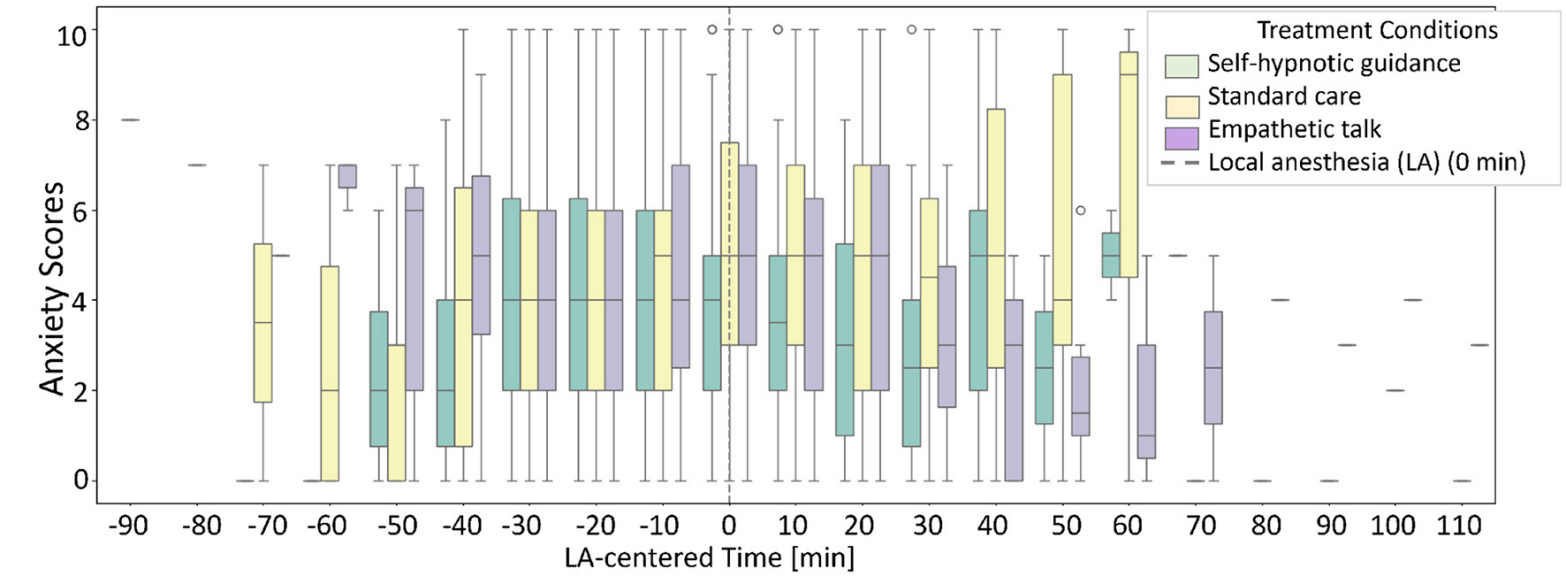
Anxiety per type of talk during biopsy. During CNB, patients received standard care (*n* = 76), patients experienced empathetic talk (*n* = 82), and patients underwent self-hypnotic talk (*n* = 78). The timing of the first local anesthesia (LA) varied for each patient.

Anxiety levels before, during, and after LA varied by intervention type: self-hypnotic, standard care, and empathetic talk (Figure 5). Both self-hypnotic and empathetic talk were associated with a post-LA decline in anxiety and shorter intervention times. Longer delays to LA were attributed to factors such as waiting time or technical complications.

Analysis of clinician word choices revealed trends in associations between median anxiety and the use of negative and positive suggestions, as well as praise. Higher total number of negative suggestions (e.g. “this will hurt”) correlated with higher median anxiety (Supplementary Figure S2A). While positive suggestions (e.g. “imagine yourself floating”) showed a slight downward trend in median anxiety (Supplementary Figure S2B). However, wide confidence intervals indicate high uncertainty. Praise (e.g. “you are doing a great job”) did not show a clear relationship with median anxiety, and the number of praises recorded was generally low across the dataset. There is considerable scatter and variability in the data.

#### 2.3.2 Insights from comments

Sentiment analysis of unstructured data were performed using the pretrained “bart-large-mnli” (BART) model (Wolf et al., 2020; Lewis et al., 2020; Kyritsis et al., 2023) that was proposed for zero-shot text classification (Yin et al., 2019; Tesfagergish et al., 2022). We chose BART due to its superior performance compared to the VADER (Hutto and Gilbert, 2014) model. Unlike VADER, which provided only positive, negative, and neutral scores, with BART we could classify texts into specific labels. From all textual comments in the database, 42,411 units of meaning and 229 unique entries were extracted, and evaluated for association with the labels “stressful,” “relaxed,” “painful,” and “painless.” Table 2 lists the top 10 comments per label, based on frequency and association value. Supplementary Table S2 details participant-specific outputs for the individuals with the maximum association value for “stressful” and “painful.” Comments with association values >0.9 were classified as “stressful” (585), “relaxed” (388), “painful” (423), and “painless” (254) and are summarized in Supplementary Data I. The automated classification provided insights into individual experience and their underlying reasons. Supplementary Figure S5 depicts components of the multifaceted CNB procedure.

**Table 2:** Extraction of top 10 comments that were classified as “painful,” “painless,” “stressful, “relaxed” by the BART model.

**Comment classified as “painful”**	**Classified as “painless”**	**Classified as “stressful”**	**Classified as “relaxed”**
Shoulder hurts	Bandaid	Very anxious	No pain
Back hurts	No bipsy taken	Discomfort	Feel fine
Arm and neck are killing me	Anesthesia I do not feel a thing	Arm hurts	Doctor very comforting
Pain from foot	No pain in breast	Stinging	Back in hypnosis
Neck and face hurting very much	Procedure was aborted so no pain	Feeling stressed	Imagery beach
Stinging incision sharp	We won't hurt you	Anxious about results	Massage neck
Very bad headache	Numb	Exhausted	Benign
Ribs are killing me	Taking no pain medication	Very tired	Happy
Sore this evening	Headache is gone	Work stress	Resting
All of a sudden I am getting pain where the needle was injected 1st time such pain	No problem sleeping	Upsetting phone call	Sleep

## 3 Discussion

We modeled the CNB process and its impact on patient experience by integrating clinical data, patient-reported outcomes, psychological pre-assessments, and workflow annotations. Although biopsies may appear to follow a standard process, our analyses revealed sub-procedures and critical events that were unique to the clinical CNB setting. The patient-centered pathway model captures different steps that patients may experience. Additionally, the predictive model for anxiety and the automated identification of experiences from patient and staff comments capture the diverse factors moderating patient experiences. Our approach profoundly extends traditional patient experience analysis, which often rely on single prospective metrics (Adams et al., 2022; Godovykh and Pizam, 2023) (e.g., Net Promoter Scores, satisfaction) and generalized service-wide measures (Adams et al., 2022).

Although our dataset included limited physiological data, we integrated patient-reported anxiety and pain before, during, and after the procedure combined with unstructured textual comments to better understand the patients' experience. Higher baseline stress was strongly associated with greater anxiety during biopsy. Pain, as defined clinically, encompasses both physical and emotional components (Chen et al., 2021; King et al., 2016). Pain, as reported by Lang et al. (2006), increased during procedural time significantly in all intervention groups (logit slopes: standard care = 0.53, empathic talk = 0.37, self-hypnosis = 0.34; all *p* < 0.001) but less steeply with self-hypnosis (*p* = 0.024) and empathetic talk (*p* = 0.018) compared to standard care. Similarly, procedural anxiety increased significantly with standard care (logit slope = 0.18, *p* < 0.001), remained stable with empathetic talk (slope = –0.04, *p* = 0.45), and decreased significantly with self-hypnosis (slope = –0.27, *p* < 0.001). Building on previous findings, our results incorporate psychological assessments and first anesthesia moment into predictive models of median anxiety.

The non-significant correlations of other variables highlight the need for larger, more diverse datasets to evaluate predictability of other psycho-social and clinical variables, including ethnicity or hormonal differences. Additionally, Bridges et al. (1991) suggests a predisposition for anxiety based on personality traits, and Smith and Pope (1992) indicate physiological distress responses vary with personality. In our dataset only textual comments complemented patients' reported anxiety and pain levels providing deeper insights into their experiences. For individuals who have difficulty to express distress, personality trait pre-assessments and sensor-based monitoring of chemical and physiological data, e.g., cortisol or heart-rate variability (Goodday and Friend, 2019; Lyzwinski et al., 2023; Singh et al., 2023), may aid in detecting distress and pain (Kumuda et al., 2018; Can et al., 2023). However, integrating corresponding data collection methods must be done carefully to avoid disrupting clinical workflows.

Our dataset lacked sufficient information to apply Lazarus psycho-emotional model for probabilistic analysis (Lazarus, 2000; Obbarius et al., 2021). Lazarus model emphasizes human self-regulation and considers the significance of the event (e.g., imminence, duration, uncertainty), alongside the ability to cope with its demands. Stressors may vary from high magnitude to low-magnitude but chronic, while coping abilities depend on both: available resources (e.g., calming atmosphere) and environmental demands (e.g., exposed breasts during procedures, or receiving care in a unfamiliar language style). Incorporating such frameworks may better account for the interplay between stressors and coping mechanisms in patient experience.

Our work underscores the complexities of medical procedures, socio-demographic, and contextual variables - many of which involve probabilities that are either unknown or not readily apparent through traditional statistical methods. As data availability increases, causal discovery techniques hold promise for identifying relationships among variables (Feuerriegel et al., 2024; Granger et al., 2024; Sanchez et al., 2022). Bayesian Networks, although resource intensive to start with, enable graphical representation of “what-if” scenarios, provide transparent estimation process, and facilitate integration of multiple outcomes in a single, cohesive model.

Our results demonstrated the critical role of unstructured, real-world textual data in modeling and analysis, particularly for understanding anxiety and pain. Although the BART model effectively identified reasons for anxiety and pain (e.g., “arm hurts”), its maximum or averaged association values were not significantly predictive of anxiety or pain in the linear mixed model. Several factors may explain the predictive limitations. First, the unstructured textual data included a mix of patient, clinician, and procedure comments, introducing variability. Second, differences in clinician roles and communication expertise-including trainee involvement or clinician-patient interaction style-may modulate patient responses (e.g., “patient not happy about trainee in the room” or “medical doctor says: concentrate on going to sleep” with the patient responding: “I'm not going to sleep!”). Third, the BART model's reliance on terms like “stressful” and “painful” may have increased sensitivity to minor stress triggers, without accounting for patients' coping mechanisms or resilience. To address these challenges, advanced natural language processing (NLP) approaches (Miller et al., 2022) are recommended, including domain-specific training (Yang et al., 2023) in medical terminology, and adaptive context-awareness to account for divergent phrasing (e.g., “Now I'll punch you” vs. “I'll take a sample now”). Finally, our analysis reveals nuanced limitations in the interpretability of NLP model outputs: the absence of comments does not necessarily exclude patient anxiety, negative staff remarks do not invariably elicit anxiety, and procedural disruptions (e.g., machine malfunction) may not always influence patient perceptions. Our findings emphasize the need for comprehensive interpretive frameworks that consider the multifaceted nature of patient-clinician interactions and procedural contexts.

The concept of a digital twin of a medical procedure, as explored in this study, offers a scalable framework for modeling complex clinical interactions and enhancing precision health. While developed in the context of breast cancer biopsy, the methodology–integrating patient-reported outcomes, psycho-social assessments, workflow annotations with clinical workflow mapping, and NLP-enabled behavioral insights–is adaptable to other invasive procedures with local anesthesia. These procedures often involve high levels of human-human interaction, where purely quantitative models fall short in capturing contextual nuances. Our approach leverages large language models (LLMs) to integrate qualitative data, enabling simulations that reflect real-life clinical care experiences. Furthermore, the predictive capabilities of these models can be embedded into real-time clinical decision support systems (CDSS). For example, real-time inputs from electronic health records (EHRs), patient-reported outcomes, and clinician annotations can be processed to identify stress points, predict adverse reactions, or recommend communication strategies. The envisioned implementation involves embedding these models into clinical workflows via interoperable platforms that support explainability and ethical safeguards (Smiee et al., 2022; El-Sappagh et al., 2021, 2023). As a first step, scalable and cost-effective digital twin modules could be developed and tested in routine clinical activities, starting with procedures where emotional and procedural complexity is high. Responsible deployment requires training clinical teams, ensuring data fairness, and protecting both patient and clinician well-being.

Although our dataset is unique, it has limitations stemming from the data collection approach and sampling frequency. The weak correlation between physiological modalities highlights the importance of future sensor-based monitoring, multi-modal sampling, and stress definition, encompassing both patient-reported measures and physiological measures. We recommend conducting power analysis and simulations to estimate data volume required for more robust predictive models.

The standard of practice, in particular, type of procedure, wait for pathology results, and educational curricula of technicians, remained mostly the same for the past 20 years. We believe that our results are relevant to large parts of the world and provide a foundational understanding of patient experience and clinical interactions during core needle biopsy procedures. Our analyses highlight critical variables and patterns that can guide the design of further investigations. In subsequent studies, patients could be supported by technologies for data collection, e.g., IoT devices, wearable sensors for stress monitoring, and data extraction from electronic health records (Guevara et al., 2024; Yang et al., 2023). Future efforts should also develop programs to modulate the patient experience within clinical data ecosystems.

Our work exemplifies how future clinical data can be collected and analyzed to monitor patient and healthcare professionals' experience, identifying critical factors of distress. Building on our models, future efforts, for example using knowledge-aware machine learning methods, should account for individual variability, procedure complexity, and real-world data constraints. Ultimately, the advancements aim to improve care experiences along the patient pathway.

## 4 Methods

### 4.1 Participants and data collection

Our study used data from the CNB clinical trial (Lang et al., 2006) conducted between February 2002 and March 2004, approved by institutional review boards of Beth Israel Deaconess Medical Center and the U.S. Army Medical Research and Materiel Command. The trail complied with Health Insurance Portability and Accountability Act (HIPAA), and participants provided written informed consent for the use of anonymized data. Inclusion criteria included female patients referred for CNB procedure attending an outpatient facility in USA, who passed screening for mental impairments (Mini Mental-State Exam) and psychosis (Schedule for Affective Disorders and Schizophrenia). Exclusion criteria included the use of oral anxiolytics or analgesics, and inability to communicate in English.

In total, 236 participants were tracked through the CNB procedure and 150 participants during a follow-up period at home. Women aged 18 to 86 years underwent ultrasound- or mammography-guided CNB and were randomized into three intervention groups: standard care (*n* = 76), empathetic talk (*n* = 82), or self-hypnotic talk (*n* = 78). Patients who underwent both, ultrasound- and mammography-guided CNB, were removed from the dataset (*n* = 3) to avoid that their extended procedure duration affects the analysis. During the follow-up period at home, participants collected salivary cortisol samples four times daily for 5 days, reporting anxiety and pain levels, and recorded diary comments. Data collection, conducted in 2002, involved manual on-site recording during CNB, video documentation of procedures, and transcription of numerical and textual values into a CSV file. The dataset includes verbal expressions from patients and clinicians. While prior analysis focused on pain progression during procedures (Lang et al., 2006), and the relationship between biopsy diagnosis uncertainty and cortisol levels (Lang et al., 2009), the current study leverages the integrated dataset to explore additional dimensions of patient experience and procedural context.

At baseline, patients provided sociodemographic data, physiological measurements, salivary cortisol levels, responses to psychological instruments, and prior biopsy experience. During the CNB procedure, patients reported pain and anxiety levels at regular intervals. A research assistant annotated procedural observations, including patient and clinician comments or situational descriptions (e.g., waiting, procedural changes, technical complications), in free-text format. Observations were documented from patient arrival through recovery, at 10-min intervals. Baseline and post-procedural salivary cortisol, heart rate, and blood pressure were recorded from monitors. Procedural data were stored per patient in a wide-format CSV file, while follow-up data were stored in long-format. Data are summarized in Supplementary Table S1. We utilized the entire dataset, including numerical and unstructured textual data to map the patient pathway (Figure 1) and derive variables which moderate the experience (Supplementary Figure S4), a published concept map for another diagnostic procedure was used as reference (Nieto Alvarez et al., 2024).

### 4.2 Data preprocessing, coding, and modeling

The dataset was labeled, organized, and uploaded into a common Python environment for descriptive analysis, variable associations, and text analysis using NLP models. Detailed code is publicly available.

Descriptive analyses included: (a) demographic trends in median anxiety, with specific analysis for ethnic or minority groups; (b) psychological instruments as predictors of anxiety; (c) associations of baseline blood pressure and heart rate with anxiety; (d) anxiety trends by type intervention type (standard care, empathetic and self-hypnotic talk); (e) associations between clinicians' (e.g., negative suggestions) and median anxiety (Supplementary Figure S2).

Unstructured textual data served as the basis for classifying sub-procedures into five types, including cases requiring additional diagnosis (e.g. surgery) or canceled procedures (Figure 1A). Sub-procedure labeling combined semi-automated categorization by computational methods with expert review. Textual annotations also identified key procedural moments, such as the first moment women received local anesthesia (LA).

We used data from 236 patients for analysis and modeling. Statistical analyses were conducted using R (v4.4.1) and the lme4 package (v1.1-29) to estimate linear mixed models. Our primary objective was to examine the effect of psychological constructs on patient-reported anxiety over time (days 1–5). Four linear mixed models were created, each analyzing one psychological construct: State-Trait Anxiety Inventory (STAI), Impact of Event Scale (IES), Center for Epidemiologic Studies Depression Scale (CES-D), and Perceived Stress Scale (PSS). Patients with fewer than three observations were excluded to ensure robust estimates. All predictors (STAI, IES, CES-D, PSS) were z-standardized, and each model included random intercepts and slopes (time) to account for individual variability. Time and procedure group were fixed effects. The dependent variable was patient-reported anxiety. The general form of the model is provided in Equation 1.


(1) $$\begin{array}{l} \begin{array}{c} Y_{ij}=\beta_{0}+\beta_{1}\times time_{ij}+\beta_{2}\times group_{i}+\beta_{3}\times X_{ij}\\ +u_{0i}+u_{1i}\times time_{ij}+\epsilon_{ij} \end{array} \end{array}$$

where:

- *Y*_*ij*_ is the patient-rated anxiety for individual *i* at time point *j*.
- β_0_ is the fixed intercept.
- β_1_ is the fixed effect of time.
- β_2_ is the fixed effect of the procedure group.
- β_3_ is the fixed effect of the z-standardized psychological construct *X*_*ij*_ which represents STAI, IES, CES-D, or PSS in separate models.
- *u*_0*i*_ is the random intercept for patient *i*, *u*_0*i*_ $\sim N_{0},\sigma_{u}^{2}$.
- *u*_1*i*_ is the random slope for time for patient *i*, *u*_1*i*_ with $\sim N_{0},\sigma_{t}^{2}$.
- ϵ_*ij*_ is the residual error term, with ϵ_*ij*_ $\sim N_{0},\sigma^{2}$.

A second linear mixed model was applied to assess whether the first time women received local anesthesia (LA) was a significant predictor of increased anxiety during CNB, identifying it as a critical moment for patients (Figure 4).

To analyze patient experience and factors derived from textual data during the procedure and subsequent 5 days, we employed NLP sentiment analysis. Comments were evaluated using the VADER model (Hutto and Gilbert, 2014) and, in a second approach, each unit of meaning evaluated for their association with the labels “painless,” “painful,” “relaxed,” and “stressful” using the pre-trained NLP model BART model (Wolf et al., 2020; Lewis et al., 2020; Kyritsis et al., 2023) designed for zero-shot text classification (Yin et al., 2019; Tesfagergish et al., 2022). The BART model calculated association values, from 0 (lowest) to 1 (highest) for each label. Comments with the highest association values (>0.9) were extracted. Maximum and averaged association values per label were computed for each participant, and they were incorporated into a linear mixed model to predict anxiety levels in the days following the procedure. To further explore individual experiences, patients with the highest association values for “painful” and “stressful” were identified and their experiences presented in Supplementary Table S2.

## Data Availability

The data analyzed in this study is subject to the following licenses/restrictions. The datasets used and analyzed during the current study are available from the corresponding author on reasonable request. Requests to access these datasets should be directed to Isabel Nieto-Alvarez, isabel.nieto@fau.de.
