# Supplementary material for: Patient-centered modeling of the breast biopsy experience
Source: Front Artif Intell. 2025 Oct 14;8:1618357. doi: 10.3389/frai.2025.1618357 (PMC12558924; doi:10.3389/frai.2025.1618357)
Supplement: Supplementary file 1 [file Data_Sheet_1.pdf]

# Supplementary Material

## 1 SUPPLEMENTARY DATA

Comments extracted as "Painful" and "Painless" by BART model with a value >0.9.

| Painful comment (BART >0.9) | Score | Painless comment (BART >0.9) | Score |
|---|---|---|---|
| all of a sudden I am getting pain when the needle was injected 1st time such pain | 0.9990 | no pain in breast | 0.9982 |
| rt . shoulder hurts | 0.9989 | anesthesia I do not feel a thing | 0.9982 |
| clipx shoulder hurts | 0.9989 | no pain | 0.9979 |
| right shoulder hurts | 0.9988 | no pain | 0.9979 |
| shoulder hurts | 0.9988 | no pain | 0.9979 |
| shoulder hurts | 0.9988 | procedure was aborted so no pain | 0.9977 |
| shoulder hurts | 0.9988 | we wont hurt you medical doctor consent | 0.9969 |
| shoulder hurts | 0.9988 | results just found out that results show no problem | 0.9966 |
| back hurts | 0.9988 | nothing painful | 0.9964 |
| back hurts | 0.9988 | so no pain | 0.9963 |
| back hurts | 0.9988 | no stinging | 0.9963 |
| back hurts | 0.9988 | scanning l side at patient request not going to hurt you | 0.9960 |
| arm neck are killing me | 0.9986 | medical doctor consent no pain | 0.9954 |
| pain from foot – trying to rework a vacation | 0.9986 | medical doctor consent : no sharp pain | 0.9952 |
| neck and face hurting very much | 0.9985 | medical doctor consent nothing sharp | 0.9950 |
| stinging incision sharp | 0.9985 | numb | 0.9944 |
| very bad headache | 0.9985 | explain clip : no extra stick | 0.9940 |
| very disappointed | 0.9984 | medical doctor prep : I promise I wont hurt you ! | 0.9939 |
| burning sting sharp | 0.9983 | no pain | 0.9931 |
| get me out of this – my arm hurts | 0.9983 | no cotton to chew | 0.9931 |
| patient : ribs are killing me | 0.9983 | should not hurt | 0.9927 |
| stinging | 0.9983 | looks pretty benign | 0.9927 |
| stinging | 0.9983 | hand numb | 0.9926 |
| arm is killing me | 0.9982 | hand numb | 0.9926 |
| sore this evening | 0.9982 | not going to hurt you | 0.9922 |
| burning and stinging dr. enters | 0.9981 | no pain | 0.9921 |
| stick x2 pain | 0.9980 | headache is gone | 0.9917 |
| stinging | 0.9980 | not pain | 0.9914 |

| | | | |
|---|---|---|---|
| stinging | 0.9980 | arm very numb | 0.9912 |
| stinging | 0.9980 | i am not anxious | 0.9912 |
| stinging | 0.9980 | taking no pain medication | 0.9907 |
| very sore | 0.9979 | easy supper | 0.9904 |
| compression hurts x | 0.9979 | nothing to biopsy | 0.9891 |
| upsetting phone call | 0.9979 | arm numb | 0.9886 |
| stings | 0.9979 | medical doctor consent : we ’re not going to hurt you | 0.9874 |
| stinging sharp | 0.9979 | no panic attacks | 0.9866 |
| holding pressure . medical doctor : this hurts the most | 0.9978 | no bleeding | 0.9866 |
| stinging burning sharp | 0.9977 | stick pain does not hurt | 0.9852 |
| again sharp pain | 0.9977 | that ’s the sharpest part patient : I do not even feel it | 0.9831 |
| left rib uncomfortable | 0.9977 | will feel warm with anesthesia no sharp pinch patient : will it hurt ? | 0.9830 |
| pinch burning and stinging | 0.9977 | results – benign | 0.9821 |
| stinging | 0.9977 | surg : benign calcs & alh | 0.9821 |
| head hurts | 0.9977 | concluded that calcs are benign ( due to vascularity ) | 0.9818 |
| head hurts | 0.9977 | benign calcs | 0.9816 |
| burns incision | 0.9976 | no problem sleeping | 0.9804 |
| burning sensation patient : it hurts | 0.9976 | no lesion found | 0.9802 |
| pinch lot of burning and stinging | 0.9976 | no lesion seen | 0.9801 |
| very bad back pain | 0.9976 | no lesion seen | 0.9801 |
| pain from dentist as novocaine wears off | 0.9976 | no lesion seen | 0.9801 |
| pinch burning stining | 0.9976 | no lesion seen | 0.9801 |
| neck is uncomfortable | 0.9976 | results word on calcifications being benign at 9:45am ! | 0.9788 |
| medical doctor need to explain stick needle pain explains complications | 0.9976 | surg : benign calcs | 0.9782 |
| terrible burning | 0.9975 | surg : benign calcs | 0.9782 |
| hurts like fire | 0.9975 | surg : benign calcs | 0.9782 |
| neck hurts massaging | 0.9975 | medical doctor : no pain consent : more noise than pain needle stick | 0.9762 |
| burn it will sting quite a bit | 0.9975 | benign | 0.9760 |
| compression hurts | 0.9974 | benign | 0.9760 |
| compression hurts | 0.9974 | benign | 0.9760 |
| xray intense pain in breast | 0.9974 | benign | 0.9760 |
| arm hurts | 0.9974 | benign | 0.9760 |
| arm hurts | 0.9974 | benign | 0.9760 |
| arm hurts | 0.9974 | benign | 0.9760 |
| arm hurts | 0.9974 | benign | 0.9760 |
| arm hurts | 0.9974 | benign | 0.9760 |

| | | | |
|---|---|---|---|
| arm hurts | 0.9974 | benign | 0.9760 |
| stinging | 0.9974 | benign | 0.9760 |
| stinging | 0.9974 | benign | 0.9760 |
| stinging | 0.9974 | benign | 0.9760 |
| neck hurts lot of pressure | 0.9974 | benign | 0.9760 |
| dental work a bit uncomfortable | 0.9973 | benign | 0.9760 |
| neck very sore | 0.9972 | no biopsy | 0.9759 |
| shoulder still sore | 0.9972 | no biopsy | 0.9759 |
| breast hurts | 0.9972 | no biopsy | 0.9759 |
| medical doctor consent lots of burning and stinging sharp | 0.9971 | no biopsy | 0.9759 |
| sting | 0.9971 | no biopsy | 0.9759 |
| sting | 0.9971 | no biopsy | 0.9759 |
| pressure hurts | 0.9971 | no biopsy | 0.9759 |
| teeny stick burn may sting | 0.9971 | no biopsy | 0.9759 |
| stinging and burning ’ | 0.9971 | no biopsy | 0.9759 |
| sore | 0.9971 | no biopsy | 0.9759 |
| sore | 0.9971 | no biopsy | 0.9759 |
| sore | 0.9971 | no biopsy | 0.9759 |
| sore | 0.9971 | no biopsy | 0.9759 |
| head hurts x | 0.9970 | no biopsy | 0.9759 |
| sting | 0.9970 | no biopsy | 0.9759 |
| sting | 0.9970 | no biopsy | 0.9759 |
| sting | 0.9970 | no biopsy | 0.9759 |
| patient : it hurts ! “ | 0.9969 | no biopsy | 0.9759 |
| neck and shoulder hurt | 0.9969 | no biopsy | 0.9759 |
| still in pain | 0.9969 | no biopsy | 0.9759 |
| that hurts | 0.9969 | no biopsy | 0.9759 |
| pain from foot | 0.9969 | no biopsy | 0.9759 |
| pain from foot | 0.9969 | no biopsy | 0.9759 |
| burning sensation everything hurts ow ! first biopsy finished | 0.9968 | no biopsy | 0.9759 |
| pressure on tissue hurts | 0.9968 | no biopsy | 0.9759 |
| had to take care of dressing on foot and shower. . . .pain is froom foot | 0.9967 | no biopsy | 0.9759 |
| burn | 0.9967 | no biopsy | 0.9759 |
| pulling stinging | 0.9967 | no biopsy | 0.9759 |
| it hurts ! “ | 0.9967 | no biopsy | 0.9759 |
| stinging and burning | 0.9967 | no biopsy | 0.9759 |
| burning ’ | 0.9966 | no biopsy | 0.9759 |
| neck hurts | 0.9966 | no biopsy | 0.9759 |
| neck hurts | 0.9966 | no biopsy | 0.9759 |
| neck hurts | 0.9966 | no biopsy | 0.9759 |
| neck hurts | 0.9966 | no biopsy | 0.9759 |
| neck hurts | 0.9966 | no biopsy | 0.9759 |

| | | | |
|---|---|---|---|
| neck hurts | 0.9966 | no biopsy | 0.9759 |
| neck hurts | 0.9966 | no biopsy | 0.9759 |
| neck hurts | 0.9966 | no biopsy | 0.9759 |
| neck hurts | 0.9966 | no biopsy | 0.9759 |
| neck hurts | 0.9966 | no biopsy | 0.9759 |
| neck hurts | 0.9966 | no biopsy | 0.9759 |
| painful | 0.9966 | no biopsy | 0.9759 |
| neck very bad - ¿ ice pack | 0.9966 | no biopsy | 0.9759 |
| medical doctor : is still stinging patient is it supposed to be ? | 0.9965 | no biopsy | 0.9759 |
| pinch burning geat samples | 0.9965 | no biopsy | 0.9759 |
| shoulder and breast pain | 0.9964 | no biopsy | 0.9759 |
| hurt | 0.9963 | no biopsy | 0.9759 |
| pressure on ribs squeezed to death | 0.9963 | no biopsy | 0.9759 |
| shoulder pain | 0.9962 | no biopsy | 0.9759 |
| shoulder pain | 0.9962 | no biopsy | 0.9759 |
| shoulder pain | 0.9962 | no biopsy | 0.9759 |
| shoulder pain | 0.9962 | no biopsy | 0.9759 |
| shoulder pain | 0.9962 | no biopsy | 0.9759 |
| head and shoulders hurt | 0.9962 | no biopsy | 0.9759 |
| neck starts hurting | 0.9962 | no biopsy | 0.9759 |
| lots of pain | 0.9962 | no biopsy | 0.9759 |
| neck is killing me anesthesia | 0.9961 | no biopsy | 0.9759 |
| some pain in left breast | 0.9961 | no biopsy | 0.9759 |
| ribs hurt | 0.9960 | no biopsy | 0.9759 |
| pain increased when bra removed | 0.9960 | no biopsy | 0.9759 |
| pain in breast increasing to 5 | 0.9959 | no biopsy | 0.9759 |
| crying because of pain | 0.9959 | no biopsy | 0.9759 |
| patient severe pain | 0.9958 | no biopsy | 0.9759 |
| most painful is my finger | 0.9958 | no biopsy | 0.9759 |
| no answer pain | 0.9958 | no biopsy | 0.9759 |
| painful to move | 0.9957 | no biopsy | 0.9759 |
| sharp pain do not move ! great | 0.9957 | no biopsy | 0.9759 |
| compression painful | 0.9956 | no biopsy | 0.9759 |
| hurts | 0.9955 | no biopsy | 0.9759 |
| sharp pain | 0.9955 | no biopsy | 0.9759 |
| sharp biopsy | 0.9954 | no biopsy | 0.9759 |
| head and neck sore | 0.9954 | no biopsy | 0.9759 |
| painful neck | 0.9954 | no biopsy | 0.9759 |
| stick sting incision | 0.9953 | no biopsy | 0.9759 |
| patient asking for more anesthesia : it hurts | 0.9953 | no biopsy | 0.9759 |
| pain at biopsy site | 0.9951 | no biopsy | 0.9759 |
| stinging many neg suggestions | 0.9950 | no biopsy | 0.9759 |
| patient : ouch ! “ | 0.9950 | no biopsy | 0.9759 |

| | | | |
|---|---|---|---|
| ow ! “ | 0.9950 | no biopsy | 0.9759 |
| burning on side of breast | 0.9949 | no induction | 0.9750 |
| foot pain | 0.9949 | medical doctor : do not wory it ’s nothing a little tylenol wo not cure procedure finished | 0.9743 |
| pinch-burn | 0.9949 | benign tissue | 0.9742 |
| sharp prick | 0.9948 | ca not take biopsy | 0.9738 |
| nech pain | 0.9947 | no biopsy necessary | 0.9726 |
| arm in pain | 0.9946 | no malignancy | 0.9724 |
| sharp pai during incision | 0.9946 | no malignancy | 0.9724 |
| may feel burning | 0.9946 | no malignancy | 0.9724 |
| very tnse – my neck hurts . have to see doc on mon . am great ! ! ! ! | 0.9945 | surg : fa with benign calcs | 0.9702 |
| very tnse – my neck hurts . have to see doc on mon . am great ! ! ! ! | 0.9945 | medical doctor in : looks like normal tissue nothing to biopsy | 0.9682 |
| patient : ow ! “ | 0.9945 | no plastic on salivette | 0.9674 |
| patient : ow ! “ | 0.9945 | tender | 0.9653 |
| patient : ow ! “ | 0.9945 | mds in & out | 0.9628 |
| soreness | 0.9945 | no biopsy taken | 0.9623 |
| only neck hurts | 0.9945 | no biopsy taken | 0.9623 |
| had pain | 0.9944 | no biopsy taken | 0.9623 |
| dull throbbing pain | 0.9944 | no biopsy taken | 0.9623 |
| sting | 0.9942 | no biopsy taken | 0.9623 |
| sting | 0.9942 | no biopsy taken | 0.9623 |
| sting | 0.9942 | no biopsy taken | 0.9623 |
| sting | 0.9942 | no biopsy taken | 0.9623 |
| pain during biopsy | 0.9940 | no biopsy taken | 0.9623 |
| neck and shoulders sore | 0.9939 | no biopsy taken | 0.9623 |
| neck hurts more than procedure | 0.9939 | no biopsy taken | 0.9623 |
| ow ! ( x5 ) | 0.9939 | no biopsy taken | 0.9623 |
| pain is from that | 0.9938 | no biopsy taken | 0.9623 |
| breast hurts ( from a lot of movement and lifting ) | 0.9938 | no biopsy taken | 0.9623 |
| intermittent pain | 0.9936 | no biopsy taken | 0.9623 |
| burning sensation | 0.9935 | no biopsy taken | 0.9623 |
| intolerable | 0.9935 | no biopsy taken | 0.9623 |
| pain | 0.9935 | no biopsy taken | 0.9623 |
| pain | 0.9935 | no biopsy taken | 0.9623 |
| arm pain | 0.9935 | no biopsy taken | 0.9623 |
| arm pain | 0.9935 | no biopsy taken | 0.9623 |
| patient refers pain from her stomach | 0.9934 | no biopsy taken | 0.9623 |
| like a bee sting ! ( tech ) | 0.9934 | no biopsy taken | 0.9623 |
| medical doctor : prick burn patient : hurts a little medical doctor : is it still stinging or is it done ? | 0.9934 | no biopsy taken | 0.9623 |

| | | | |
|---|---|---|---|
| hurt and pain | 0.9934 | no biopsy taken | 0.9623 |
| sharp pinch | 0.9933 | no biopsy taken | 0.9623 |
| sting | 0.9932 | no biopsy taken | 0.9623 |
| sting | 0.9932 | no biopsy taken | 0.9623 |
| anesthesia hurts | 0.9931 | no biopsy taken | 0.9623 |
| painfull uss | 0.9931 | no biopsy taken | 0.9623 |
| neck hurts more | 0.9929 | looks benign | 0.9615 |
| shoulders hurt | 0.9929 | no histology at bidmc | 0.9609 |
| headache this morning | 0.9929 | pain lessened | 0.9602 |
| patient very uncomfortable on table | 0.9928 | no answer pain | 0.9562 |
| head-ache | 0.9927 | 98 % benign | 0.9507 |
| sore breast | 0.9925 | should not feel sharp | 0.9505 |
| sore breast | 0.9925 | prob benign | 0.9501 |
| area hurts when touched | 0.9924 | surg : no mass | 0.9494 |
| saw – foot aches | 0.9923 | no tumour | 0.9475 |
| soreness going | 0.9922 | too tired to feel anxious | 0.9434 |
| burning | 0.9921 | feel little pain only while squeezing ( breast examining ) | 0.9429 |
| burning | 0.9921 | benign parenchyma | 0.9427 |
| burning | 0.9921 | no core biopsy | 0.9400 |
| burning | 0.9921 | scans ( no needle yet ) | 0.9381 |
| burning | 0.9919 | benign adenosis | 0.9345 |
| only pressure hurts | 0.9917 | no lump found | 0.9345 |
| arthritis pain | 0.9917 | no biopsy taken as simple cyst on uss | 0.9331 |
| arthritis pain | 0.9917 | patient left alone | 0.9288 |
| patient pain | 0.9916 | very relaxd | 0.9281 |
| arm hurt | 0.9915 | ice pack cold – feeling better | 0.9257 |
| still bleedin g | 0.9914 | will not biopsy | 0.9233 |
| chronic neck pain | 0.9912 | feel fine | 0.9211 |
| pain in ribs | 0.9911 | feel fine | 0.9211 |
| upset since 4pm | 0.9910 | feel fine | 0.9211 |
| arm very uncomfortable | 0.9909 | should not feel pain or sharp | 0.9192 |
| burned myself on arm cooking | 0.9906 | beach imagery quiet | 0.9170 |
| bleeding ++ with biopsy | 0.9906 | sterile drape | 0.9158 |
| ow ! medical doctor : is that still stining ? “ | 0.9905 | no tumor seen | 0.9155 |
| neck pain | 0.9905 | band-aid | 0.9094 |
| neck pain | 0.9905 | band-aid | 0.9094 |
| neck pain | 0.9905 | band-aid | 0.9094 |
| neck pain | 0.9905 | band-aid | 0.9094 |
| neck pain | 0.9905 | band-aid | 0.9094 |
| sore neck due to positioning | 0.9904 | band-aid | 0.9094 |
| sore from shower | 0.9904 | band-aid | 0.9094 |

| | | | |
|---|---|---|---|
| left arm sore | 0.9902 | band-aid | 0.9094 |
| left arm sore | 0.9902 | band-aid | 0.9094 |
| back pain | 0.9900 | band-aid | 0.9094 |
| back pain | 0.9900 | band-aid | 0.9094 |
| back pain | 0.9900 | band-aid | 0.9094 |
| back pain | 0.9900 | band-aid | 0.9094 |
| back pain | 0.9900 | band-aid | 0.9094 |
| back pain | 0.9900 | band-aid | 0.9094 |
| back pain | 0.9900 | band-aid | 0.9094 |
| itching . pulled off over bandage . saw breast . thinking . praying | 0.9897 | band-aid | 0.9094 |
| burn | 0.9894 | band-aid | 0.9094 |
| capsule tasted foul | 0.9890 | band-aid | 0.9094 |
| pain pressure | 0.9890 | band-aid | 0.9094 |
| breast pain | 0.9890 | band-aid | 0.9094 |
| breast pain | 0.9890 | band-aid | 0.9094 |
| bleeding | 0.9888 | band-aid | 0.9094 |
| bleeding | 0.9888 | band-aid | 0.9094 |
| bleeding | 0.9888 | band-aid | 0.9094 |
| bleeding | 0.9888 | band-aid | 0.9094 |
| bleeding | 0.9888 | band-aid | 0.9094 |
| bleeding | 0.9888 | band-aid | 0.9094 |
| bleeding | 0.9888 | band-aid | 0.9094 |
| bleeding | 0.9888 | gut feeling it is benign | 0.9063 |
| bleeding | 0.9888 | so low anxiety | 0.9050 |
| bleeding | 0.9888 | no biopsy on right side | 0.9028 |
| bleeding | 0.9888 | tech in and out | 0.9020 |
| bleeding | 0.9888 | | |
| bleeding | 0.9888 | | |
| bleeding | 0.9888 | | |
| anesthesia sting burn beautiful needle in | 0.9887 | | |
| feels punching | 0.9887 | | |
| is it still stinging pinching | 0.9886 | | |
| neck very bad | 0.9885 | | |
| prep area prick & burning sensation | 0.9885 | | |
| patient feeling burning | 0.9882 | | |
| breast uncomfortable | 0.9881 | | |
| patient leg discomfort | 0.9880 | | |
| shoulder sore | 0.9875 | | |
| shoulder sore | 0.9875 | | |
| shoulder sore | 0.9875 | | |
| knee discomfort | 0.9873 | | |
| body pain | 0.9871 | | |
| hurts more anesthesia | 0.9871 | | |

| | | | |
|---|---|---|---|
| back discomfort | 0.9870 | | |
| hard to get up | 0.9869 | | |
| vicadan for pain | 0.9866 | | |
| stung in finger by yellow jacket at 7.15pm . pain and swelling . pain unrelated to biopsy | 0.9866 | | |
| patient very uncomfortable | 0.9865 | | |
| had a headache | 0.9861 | | |
| needle in pain | 0.9860 | | |
| tearful | 0.9858 | | |
| pain | 0.9856 | | |
| pain | 0.9856 | | |
| sharp needle in | 0.9854 | | |
| arm sore | 0.9852 | | |
| arm sore | 0.9852 | | |
| arm sore | 0.9852 | | |
| dr. explains gopal pain sharp very good am I a difficult patient ? no . beautiful | 0.9852 | | |
| took a pain pill and going ot bi to find out results – did not sleep at all last night | 0.9850 | | |
| headache | 0.9844 | | |
| headache | 0.9844 | | |
| headache | 0.9844 | | |
| headache | 0.9844 | | |
| have stomach ache | 0.9844 | | |
| have stomach ache | 0.9844 | | |
| burn | 0.9843 | | |
| burn | 0.9843 | | |
| want to cry almost crying | 0.9843 | | |
| achy all over | 0.9841 | | |
| feel sharp | 0.9837 | | |
| much more uncomfortable with recentering | 0.9833 | | |
| patient disappointed | 0.9830 | | |
| patient disappointed | 0.9830 | | |
| patient can hardly breathe | 0.9829 | | |
| still some back discomfort | 0.9826 | | |
| neck terrible | 0.9826 | | |
| ribs slightly sore - wants to go home | 0.9823 | | |
| patient wish I could move my leg | 0.9813 | | |
| aggrevation | 0.9811 | | |
| weird that I still have pain | 0.9807 | | |
| soreness in breast | 0.9806 | | |

| | | | |
|---|---|---|---|
| sharp | 0.9799 | | |
| sharp | 0.9799 | | |
| sharp | 0.9799 | | |
| sharp | 0.9799 | | |
| sharp | 0.9799 | | |
| sharp | 0.9799 | | |
| sharp | 0.9799 | | |
| sharp | 0.9799 | | |
| wound is now itchy | 0.9797 | | |
| a lot off bleeding | 0.9790 | | |
| needle stick patient : do not like needles | 0.9775 | | |
| only hurts when I move a lot | 0.9775 | | |
| car problems – what a week ! | 0.9773 | | |
| patient alone again ( headache 2 days ) | 0.9771 | | |
| pissed off again at my situation | 0.9758 | | |
| needle stick | 0.9757 | | |
| needle stick | 0.9757 | | |
| needle stick | 0.9757 | | |
| needle stick | 0.9757 | | |
| needle prick | 0.9755 | | |
| feeling pinch | 0.9753 | | |
| i know the results are bad | 0.9746 | | |
| feel pain only when doing sharp movement with my right arm ( right breast ) | 0.9744 | | |
| burning ? “ | 0.9743 | | |
| patient feels needle | 0.9741 | | |
| you ’ll feel it going in . uncomfortable for a few seconds | 0.9740 | | |
| some lower back pain | 0.9737 | | |
| 2abscess dr | 0.9734 | | |
| felt sharp on last biopsy | 0.9726 | | |
| it ’s really sharp | 0.9714 | | |
| patient screams while inserting clip | 0.9713 | | |
| inner samples were uncomfortable | 0.9701 | | |
| medical doctor : pain ? pinching ? “ | 0.9690 | | |
| felt some burning on 3rd | 0.9685 | | |
| sharp x | 0.9678 | | |
| a little back pain | 0.9673 | | |
| patient is bleeding a lot | 0.9672 | | |
| patient : ow ! : medical doctor : that ’s just the local going in | 0.9668 | | |
| yankees losing to marlins | 0.9665 | | |

| | | | |
|---|---|---|---|
| family on my nerves | 0.9663 | | |
| discomfort of the table | 0.9660 | | |
| discomfort of the table | 0.9660 | | |
| small needle stick sorry for the pressure | 0.9660 | | |
| medical doctor pushing on your breast | 0.9658 | | |
| ressure | 0.9657 | | |
| bruised | 0.9642 | | |
| was bleeding | 0.9632 | | |
| upset | 0.9632 | | |
| menstral cramps | 0.9628 | | |
| gh discomfort | 0.9625 | | |
| crying to start with | 0.9622 | | |
| lots of bleeding | 0.9614 | | |
| patient very upset | 0.9613 | | |
| bruising appeared | 0.9599 | | |
| tech : does that sting ? “ | 0.9592 | | |
| position uncomfortable | 0.9584 | | |
| neck uncomfortable | 0.9583 | | |
| neck uncomfortable | 0.9583 | | |
| results – maligt | 0.9579 | | |
| results – maligt | 0.9579 | | |
| medical doctor : is it still stinging ? needle in | 0.9570 | | |
| i ’ll go mad ! I was hoping tomorrow ! | 0.9567 | | |
| lesion not well visualized | 0.9563 | | |
| patient : ow medical doctor : feel some pain ? | 0.9562 | | |
| stomach upset | 0.9556 | | |
| arm uncomfortable | 0.9518 | | |
| discomfort | 0.9513 | | |
| discomfort | 0.9513 | | |
| discomfort | 0.9513 | | |
| discomfort | 0.9513 | | |
| discomfort | 0.9513 | | |
| discomfort | 0.9513 | | |
| position is uncomfortabl | 0.9501 | | |
| medical doctor : sharp ? patient : that was prickly | 0.9501 | | |
| pain pill | 0.9497 | | |
| pain pill | 0.9497 | | |
| can not continue | 0.9475 | | |
| still nauseous | 0.9473 | | |

| | | | |
|---|---|---|---|
| still nauseous | 0.9473 | | |
| asthma is starting | 0.9463 | | |
| uss pinch | 0.9457 | | |
| discomfort ( not breast ) | 0.9449 | | |
| had a large blister lanced at 2 | 0.9435 | | |
| upset waiting for medical doctor | 0.9432 | | |
| then disappear patient : ow ! medical doctor : should not feel sharp | 0.9420 | | |
| these are cyts . do you really want us to stick you with needles ? | 0.9414 | | |
| nobody listens | 0.9409 | | |
| patient felt needle | 0.9402 | | |
| 2 hours waiting time ! ! ! | 0.9398 | | |
| still tender | 0.9396 | | |
| patient used to pain daily as arthritic ++ | 0.9373 | | |
| angry that dr is not doing it herself | 0.9369 | | |
| very difficult for to get angle | 0.9367 | | |
| prick | 0.9366 | | |
| prick | 0.9366 | | |
| patient : ow medical doctor : feel a pinch there ? | 0.9352 | | |
| medical doctor : use ice to help with pain | 0.9335 | | |
| patient frustrated – wishes they 'd taken care of this beforehand | 0.9292 | | |
| patient said back pain was worse than pain from procedure | 0.9286 | | |
| positioning pinch prick | 0.9250 | | |
| results negative ! | 0.9237 | | |
| a little sore | 0.9225 | | |
| situation a bit too much for her | 0.9212 | | |
| talking family busness issues – upset about them | 0.9209 | | |
| pinch | 0.9203 | | |
| if it hurts | 0.9177 | | |
| irritating eyes | 0.9154 | | |
| results neg results ! | 0.9106 | | |
| suffering from severe seasonal allergies | 0.9093 | | |
| suffering from severe seasonal allergies | 0.9093 | | |
| talking about dead husband | 0.9076 | | |
| patient discomfort due to positioning | 0.9064 | | |

| | | | |
|---|---|---|---|
| little sting | 0.9053 | | |
| medical doctor explains procedure | 0.9027 | | |

Comments extracted as “Stressful” and “Relaxed” by BART model with a value >0.9.

| **Stressful comment (BART >0.9)** | **Score** | **Relaxed comment (BART >0.9)** | **Score** |
|---|---|---|---|
| about to give a reception for 55 people : stress | 0.9994 | just had nap | 0.999497 |
| upsetting phone call | 0.9991 | very calm | 0.999278 |
| situation a bit too much for her | 0.9988 | patient resting comfortably | 0.998829 |
| very tense | 0.9986 | patient resting comfortably | 0.998829 |
| very tense | 0.9986 | benign | 0.998321 |
| dr very hectic | 0.9985 | benign | 0.998321 |
| get me out of this – my arm hurts | 0.9984 | benign | 0.998321 |
| medical doctor very harried | 0.9982 | benign | 0.998321 |
| very anxious | 0.9979 | benign | 0.998321 |
| very anxious | 0.9979 | benign | 0.998321 |
| very anxious | 0.9979 | benign | 0.998321 |
| very anxious | 0.9979 | benign | 0.998321 |
| very anxious | 0.9979 | benign | 0.998321 |
| very anxious | 0.9979 | benign | 0.998321 |
| very nervous because tech told her that biopsy maybe not necessary | 0.9978 | benign | 0.998321 |
| feeling very anxious work and biopsy combined | 0.9977 | benign | 0.998321 |
| anxiety level is because of not knowing the results of biopsy yet | 0.9977 | benign | 0.998321 |
| dental work a bit uncomfortable | 0.9974 | benign | 0.998321 |
| feeling overwhelmed with workload | 0.9973 | benign | 0.998321 |
| too risky position to biopsy | 0.9972 | no problem sleeping | 0.998197 |
| call from doctor triggered anxiety | 0.9972 | i am not anxious | 0.998167 |
| feels very tense | 0.9971 | looks pretty benign | 0.998051 |
| feeling pretty anxious | 0.9969 | relaxing | 0.997917 |
| car problems – what a week ! | 0.9969 | very relaxed at beach | 0.997853 |
| busy of course anxiety and then some | 0.9969 | on mini vacation | 0.997816 |
| tense | 0.9968 | relaxed | 0.997769 |
| pressure hurts | 0.9967 | patient resting | 0.997765 |
| family on my nerves | 0.9967 | patient resting | 0.997765 |
| i ’m a nervous wreck | 0.9965 | patient resting | 0.997765 |
| patient can hardly breathe | 0.9965 | very relieved | 0.997739 |
| work anxiety | 0.9963 | patient very calm | 0.997729 |
| hard day with work load . got through it | 0.9960 | looks benign | 0.997664 |

| Stressful comment (BART >0.9) | Score | Relaxed comment (BART >0.9) | Score |
|---|---|---|---|
| stings | 0.9960 | took a nap | 0.99749 |
| arm & neck are killing me | 0.9959 | drove from hospital to central square for dinner and relaxing | 0.997439 |
| anxious | 0.9959 | sleeping | 0.997357 |
| neck is uncomfortable | 0.9958 | sleeping | 0.997357 |
| stinging | 0.9957 | easy supper | 0.997152 |
| busy morning | 0.9957 | beach imagery quiet | 0.997073 |
| work pressure | 0.9957 | i 'll fall asleep relaxed | 0.996971 |
| compression painful | 0.9956 | said she does well with this kind of stuff was not very anxious | 0.996967 |
| upset since 4pm | 0.9955 | feeling sleepy | 0.99689 |
| life was to stressful right now | 0.9955 | rested and relaxed | 0.996883 |
| upset waiting for medical doctor | 0.9954 | and am not at all thinking about the lump . it is beautiful friday night and we had a bbq outside as the sun set | 0.996822 |
| pressure on ribs squeezed to death | 0.9953 | patient dozing | 0.996776 |
| probably more anxiety after the test than during because I assumed this was all very precautionary and then the doctor reminded me to call friday for results . results ? ! I hope none ! | 0.9953 | sleepy | 0.996765 |
| anxious re results | 0.9952 | very relaxed | 0.996718 |
| pressure on tissue hurts | 0.9951 | resting | 0.996606 |
| office angst | 0.9951 | very relaxd | 0.996564 |
| small needle stick sorry for the pressure | 0.9951 | i 'm okay | 0.996557 |
| anxious about results | 0.9951 | she makes me calm | 0.996469 |
| anxious about results | 0.9951 | feeling sleepy | 0.996419 |
| pissed off again at my situation | 0.9950 | results – benign | 0.996273 |
| dr makes patient nervous with several neg suggestions | 0.9950 | relaxed atmosphere | 0.996232 |
| anxiety elevated due to lack of results | 0.9949 | up from a nap | 0.996051 |
| compression hurts x | 0.9948 | feel fine | 0.996026 |
| very nervous | 0.9946 | feel fine | 0.996026 |
| very nervous | 0.9946 | feel fine | 0.996026 |
| i have a very busy day today | 0.9946 | so low anxiety | 0.995942 |
| sharp pain do not move ! great | 0.9945 | patient sleeping | 0.995787 |
| feeling stressed | 0.9944 | patient sleeping | 0.995787 |
| feeling stressed | 0.9944 | patient sleeping | 0.995787 |
| slight anxiety attack | 0.9944 | slept well | 0.995555 |
| patient frustrated – wishes they 'd taken care of this beforehand | 0.9944 | slept well | 0.995555 |
| patient : im nervous | 0.9944 | slept well | 0.995555 |

| Stressful comment (BART >0.9) | Score | Relaxed comment (BART >0.9) | Score |
|---|---|---|---|
| arm is killing me | 0.9944 | feels relaxed | 0.995464 |
| anxious and angry none informed her in a better way | 0.9943 | patient stays calm | 0.995321 |
| became a little anxious at work today | 0.9943 | hand falling asleep | 0.994794 |
| anxiety increasing | 0.9943 | at beach feeling wonderful | 0.99476 |
| worried about lay-offs for my staff | 0.9943 | patient left alone | 0.994755 |
| irritating eyes | 0.9942 | slept late | 0.994493 |
| patient : it hurts ! “ | 0.9942 | patient not anxious | 0.99447 |
| it hurts ! “ | 0.9942 | 98 % benign | 0.994255 |
| nervous | 0.9942 | loking forward to sleep | 0.994185 |
| nervous | 0.9942 | low anxiety but still awaiting test results . expecting negative results | 0.994147 |
| compression hurts | 0.9941 | was still sleeping | 0.994134 |
| compression hurts | 0.9941 | was still sleeping | 0.994134 |
| am starting to get a bit of anxiety in my stomach because I have to go to work | 0.9940 | patient very relaxed after procedure | 0.994096 |
| my anxiety | 0.9938 | relaxed | 0.99403 |
| hurts | 0.9938 | not anxious about procedure | 0.993928 |
| patient felt pressure | 0.9938 | arm falling asleep | 0.993732 |
| been rushing around all day – always late ! | 0.9937 | patient calming down | 0.993575 |
| neck hurts lot of pressure | 0.9937 | not anxious now – anxiety will come while I ’m waiting for results | 0.993372 |
| work stress | 0.9936 | not concerned about proc | 0.993372 |
| work stress | 0.9936 | probably benign | 0.993291 |
| work stress | 0.9936 | tender | 0.993084 |
| stinging | 0.9936 | prob benign | 0.992942 |
| stinging | 0.9936 | at this time I do not have much anxiety . a bit of a stiff neck | 0.992774 |
| feeling pressure | 0.9935 | smiling | 0.99274 |
| work pressure and personal | 0.9934 | it ’ll be fine | 0.992266 |
| took a pain pill and going ot bi to find out results – did not sleep at all last night | 0.9934 | too tired to feel anxious | 0.991939 |
| burning sensation everything hurts ow ! first biopsy finished | 0.9933 | not thinking about lump | 0.991829 |
| medical doctor should have anxiety | 0.9933 | benign calcs | 0.991631 |
| all of a sudden scared . my cousin freaked me out at dinner . I do not want to have cancer | 0.9933 | hand numb | 0.991627 |
| patient very uncomfortable | 0.9932 | hand numb | 0.991627 |
| no time for anxiety | 0.9931 | patient relaxed ( knows how to use self-hypnosis she said before ) | 0.991606 |

| Stressful comment (BART >0.9) | Score | Relaxed comment (BART >0.9) | Score |
|---|---|---|---|
| stinging many neg suggestions | 0.9931 | gut feeling it is benign | 0.991375 |
| too busy | 0.9930 | just sat down and had a bite to eat and was by myself | 0.991051 |
| sting | 0.9930 | will feel cool | 0.9905 |
| sting | 0.9930 | patient feels relaxed | 0.989955 |
| sting | 0.9930 | arm numb | 0.98983 |
| patient crying because of anxiety | 0.9930 | patient sleepy | 0.989772 |
| only pressure hurts | 0.9928 | anesthesia I do not feel a thing | 0.989457 |
| very difficult for dr. to get angle | 0.9927 | ca not imagine herself being elsewhere | 0.989439 |
| patient irritated attempting to find viable angle for biopsy | 0.9927 | patient relieved | 0.989434 |
| hurts like fire | 0.9926 | great relief ’ | 0.989324 |
| work-related stress | 0.9926 | results just found out that results show no problem | 0.989292 |
| anxious about the results | 0.9926 | is this test relevant now ? I just called in for my result – which thankfully were fine . so I feel calm | 0.989094 |
| stinging | 0.9925 | off work | 0.988968 |
| stinging | 0.9925 | saturday ( no call ? ) | 0.988545 |
| stinging | 0.9925 | we wo not hurt you medical doctor consent | 0.988385 |
| talking family busness issues – upset about them | 0.9925 | feel tired but fine | 0.988372 |
| got nervous after discussion of when results would be known | 0.9925 | saw breast showering . when not busy | 0.988094 |
| pain pressure | 0.9925 | on tranquellizers | 0.987964 |
| my kids are driving me crazy ! | 0.9924 | patient meditating | 0.987729 |
| stinging burning sharp | 0.9922 | patient somewhat relieved | 0.987445 |
| pain from foot – trying to rework a vacation | 0.9922 | just want a nice relaxing summer weekend | 0.986802 |
| painfull uss | 0.9922 | benign tissue | 0.986763 |
| burning and stinging dr. b enters | 0.9922 | scanning l side at patient request not going to hurt you | 0.986734 |
| position is uncomfortabl | 0.9921 | patient felt v calm after the procedure | 0.986284 |
| stinging and burning ’ | 0.9918 | in a good mood | 0.986066 |
| worried about if it will start to bleed again and what to do | 0.9918 | benign parenchyma | 0.98577 |
| anxiety from | 0.9918 | medical doctor consent : we ’re not going to hurt you | 0.985752 |
| patient : ribs are killing me | 0.9916 | should not hurt | 0.985745 |
| then disappear patient : ow ! medical doctor : should not feel sharp | 0.9915 | well distracted by guests and errands . warm summer day | 0.985181 |

| Stressful comment (BART >0.9) | Score | Relaxed comment (BART >0.9) | Score |
|---|---|---|---|
| anxious re what happens next | 0.9913 | focused on breathing | 0.984884 |
| painful neck | 0.9912 | late dinner | 0.984809 |
| upset | 0.9912 | everyone leaves room | 0.984272 |
| shaky | 0.9911 | waiting | 0.983721 |
| patient : ow ! “ | 0.9910 | waiting | 0.983721 |
| patient : ow ! “ | 0.9910 | waiting | 0.983721 |
| patient : ow ! “ | 0.9910 | waiting | 0.983721 |
| job stress only at work | 0.9910 | waiting | 0.983721 |
| fatigued | 0.9909 | waiting | 0.983721 |
| fatigued | 0.9909 | waiting | 0.983721 |
| fatigued | 0.9909 | waiting | 0.983721 |
| i ’ll go mad ! I was hoping tomorrow ! | 0.9909 | waiting | 0.983721 |
| pressure | 0.9909 | waiting | 0.983721 |
| pressure | 0.9909 | found relaxation v helpful | 0.983342 |
| pressure | 0.9909 | patient closed eyes | 0.981891 |
| pressure | 0.9909 | found relaxation very helpful | 0.981561 |
| pressure | 0.9909 | medical doctor consent nothing sharp | 0.98145 |
| pressure | 0.9909 | medical doctor very comforting | 0.980405 |
| pressure | 0.9909 | feel great god is with me | 0.980111 |
| pressure | 0.9909 | on the beach with family | 0.980058 |
| pressure | 0.9909 | ca not take biopsy | 0.979446 |
| pressure | 0.9909 | patient wants to sleep | 0.979416 |
| pressure | 0.9909 | patient wants to sleep | 0.979416 |
| pressure | 0.9909 | no pain ’ | 0.978984 |
| pressure | 0.9909 | having a good time | 0.977976 |
| hard to get up | 0.9908 | dr k very comforting | 0.977653 |
| little nervous wants ’valium ( does not get it of course ) | 0.9907 | dr k very comforting | 0.977653 |
| stinging sharp | 0.9906 | i feel terrific | 0.976762 |
| just wish they ’d get it overwith | 0.9905 | will not biopsy | 0.97641 |
| worried re | 0.9905 | await doctor call – not bad news | 0.975565 |
| patient very uncomfortable on table | 0.9904 | i feel great | 0.975289 |
| but anxious about results | 0.9904 | numb | 0.975132 |
| patient very upset | 0.9904 | arm very numb | 0.975107 |
| medical doctor to patient want more anesthesia ? you ’re the boss ! sorry for the pressure | 0.9904 | breast fine | 0.974921 |
| slept bad again – starting to stress a little | 0.9901 | no biopsy taken | 0.974903 |
| stinging | 0.9900 | no biopsy taken | 0.974903 |
| stinging | 0.9900 | no biopsy taken | 0.974903 |
| stinging | 0.9900 | no biopsy taken | 0.974903 |

| Stressful comment (BART >0.9) | Score | Relaxed comment (BART >0.9) | Score |
|---|---|---|---|
| stinging | 0.9900 | no biopsy taken | 0.974903 |
| patient crying & shaking | 0.9900 | no biopsy taken | 0.974903 |
| celebrage negative results ! very tired | 0.9900 | no biopsy taken | 0.974903 |
| sting | 0.9899 | no biopsy taken | 0.974903 |
| sting | 0.9899 | no biopsy taken | 0.974903 |
| pressure | 0.9899 | no biopsy taken | 0.974903 |
| patient worried about surgery | 0.9897 | no biopsy taken | 0.974903 |
| stinging and burning | 0.9896 | no biopsy taken | 0.974903 |
| pressure feeling on top of head | 0.9895 | no biopsy taken | 0.974903 |
| medical doctor gives patient the feeling of study as a disturbing factor | 0.9895 | no biopsy taken | 0.974903 |
| restless | 0.9894 | no biopsy taken | 0.974903 |
| rushing | 0.9893 | no biopsy taken | 0.974903 |
| patient frightened samples | 0.9891 | no biopsy taken | 0.974903 |
| all of a sudden I am getting pain when the needle was injected 1st time such pain | 0.9891 | no biopsy taken | 0.974903 |
| may feel burning | 0.9890 | no biopsy taken | 0.974903 |
| very bad headache | 0.9889 | no biopsy taken | 0.974903 |
| painful to move | 0.9887 | no biopsy taken | 0.974903 |
| anxiety over daily routine ! large party this weekend | 0.9887 | no biopsy taken | 0.974903 |
| patient screams while inserting clip | 0.9886 | no biopsy taken | 0.974903 |
| arm very uncomfortable | 0.9885 | no biopsy taken | 0.974903 |
| uss pinch | 0.9884 | no biopsy taken | 0.974903 |
| physician consent increased anxiety initially | 0.9883 | no biopsy taken | 0.974903 |
| patient very nervous | 0.9882 | no biopsy taken | 0.974903 |
| busy – oops late again ! | 0.9879 | no biopsy taken | 0.974903 |
| patient feeling burning | 0.9877 | no biopsy taken | 0.974903 |
| ow ! medical doctor : is that still stining ? “ | 0.9875 | no biopsy taken | 0.974903 |
| very tnse – my neck hurts . have to see doc on mon . am great ! ! ! ! | 0.9873 | results word on calcifications being benign at 9:45am ! | 0.974826 |
| very tnse – my neck hurts . have to see doc on mon . am great ! ! ! ! | 0.9873 | fluid was nondiagnostic | 0.974342 |
| still tired ( cleaning ) | 0.9871 | surg : benign calcs | 0.974328 |
| dr. messed it up again - ¿ wrong incision - ¿ start all over again | 0.9868 | surg : benign calcs | 0.974328 |
| holding pressure . medical doctor : this hurts the most | 0.9867 | surg : benign calcs | 0.974328 |
| startled patient | 0.9867 | medical doctor consent no pain | 0.974218 |

| Stressful comment (BART >0.9) | Score | Relaxed comment (BART >0.9) | Score |
|---|---|---|---|
| clipshoulder hurts | 0.9866 | nothing to biopsy | 0.973972 |
| no answer pain | 0.9866 | patient ca not imagine place but feels relaxed | 0.973636 |
| had a headache | 0.9866 | feeling a little better | 0.973605 |
| anxiety from family | 0.9865 | so no pain | 0.973495 |
| rushing to prepare brunch for guests | 0.9864 | no pain | 0.973477 |
| much more uncomfortable with recentering | 0.9863 | nothing painful | 0.97337 |
| achy all over | 0.9863 | mds leave patient alone with provider | 0.97336 |
| head and shoulders hurt | 0.9862 | no panic attacks | 0.973018 |
| ow ! “ | 0.9860 | patient very relieved . | 0.972998 |
| patient asking for more anesthesia : it hurts | 0.9857 | therefore no biopsy taken | 0.971118 |
| anxious night sleep | 0.9856 | i am not awake | 0.971101 |
| stinging incision sharp | 0.9855 | not going to hurt you | 0.970721 |
| head hurts x | 0.9855 | imagery of warm bath | 0.970607 |
| worried about test results | 0.9853 | medical doctor states that biopsy is probably not warranted – fibroadenoma likely | 0.970562 |
| worried about results | 0.9848 | medical doctor in : looks like normal tissue nothing to biopsy | 0.969775 |
| terrible burning | 0.9848 | allowed to move | 0.969144 |
| stick x2 pain | 0.9847 | dr. k tells patient they probably ca not do biopsy | 0.968214 |
| sting | 0.9847 | short babysitting – grandchildren are delicious ! | 0.967526 |
| sting | 0.9847 | am okay | 0.967385 |
| sting | 0.9847 | headache is gone | 0.966068 |
| sting | 0.9847 | we are gon na do this | 0.965733 |
| long delay waiting for radiologist after uss while in hypnosis | 0.9846 | no cotton to chew | 0.965001 |
| stress with kids | 0.9843 | ice pack cold – feeling better | 0.964055 |
| history of panic attacks | 0.9843 | back in hypnosis | 0.963241 |
| medical doctor : is still stinging patient is it supposed to be ? | 0.9840 | back in hypnosis | 0.963241 |
| hof panic attacks | 0.9840 | surg : fa with benign calcs | 0.962363 |
| pinch burning and stinging | 0.9837 | not anxious just sad | 0.962279 |
| patient realizing biopsy will actually happen | 0.9835 | the more you relathe better you ’ll feel ! | 0.962272 |
| patient upset | 0.9835 | not feeling discomfort – just annoyance | 0.961945 |
| patient scared | 0.9835 | you are doing fine | 0.961649 |

| Stressful comment (BART >0.9) | Score | Relaxed comment (BART >0.9) | Score |
|---|---|---|---|
| renal cancer treatment at bidmc – patient gets nauseous when I enter the building | 0.9830 | not as anxious as I thought I 'll be | 0.961186 |
| head hurts | 0.9829 | feel better | 0.961085 |
| head hurts | 0.9829 | patient discussed experience of relaxation | 0.960867 |
| irritated by coworker | 0.9829 | no biopsy | 0.960734 |
| anxiety disorder | 0.9828 | no biopsy | 0.960734 |
| hurt | 0.9823 | no biopsy | 0.960734 |
| again sharp pain | 0.9823 | no biopsy | 0.960734 |
| nervous about test results | 0.9816 | no biopsy | 0.960734 |
| very tired today | 0.9816 | no biopsy | 0.960734 |
| pressure band-aid | 0.9815 | no biopsy | 0.960734 |
| pressure band-aid | 0.9815 | no biopsy | 0.960734 |
| pressure band-aid | 0.9815 | no biopsy | 0.960734 |
| looking for this vial – hunted all over house | 0.9814 | no biopsy | 0.960734 |
| ow ! ( x5 ) | 0.9813 | no biopsy | 0.960734 |
| patient yelling at nurse | 0.9812 | no biopsy | 0.960734 |
| xray intense pain in breast | 0.9812 | no biopsy | 0.960734 |
| results angry – why me ? ! | 0.9807 | no biopsy | 0.960734 |
| position uncomfortable | 0.9804 | no biopsy | 0.960734 |
| sting | 0.9803 | no biopsy | 0.960734 |
| sting | 0.9803 | no biopsy | 0.960734 |
| starts getting worried about possible surgery | 0.9803 | no biopsy | 0.960734 |
| angry that dr is not doing it herself | 0.9803 | no biopsy | 0.960734 |
| forgot to do it... driving in the snow for 2 hours ! ! | 0.9801 | no biopsy | 0.960734 |
| running late on this time .. rainy day . next off to work | 0.9801 | no biopsy | 0.960734 |
| want to cry almost crying | 0.9800 | no biopsy | 0.960734 |
| patient shaking | 0.9798 | no biopsy | 0.960734 |
| long day lots of meetings in the afternoon | 0.9792 | no biopsy | 0.960734 |
| late for work ! | 0.9792 | no biopsy | 0.960734 |
| thinking about biopsy results and how long I have to wait | 0.9790 | no biopsy | 0.960734 |
| neck and face hurting very much | 0.9789 | no biopsy | 0.960734 |
| anxious about time | 0.9788 | no biopsy | 0.960734 |
| feeling a bit anxious this morning | 0.9787 | no biopsy | 0.960734 |
| some anxiety when I saw the wound after taking the bandage off | 0.9787 | no biopsy | 0.960734 |
| medical doctor consent lots of burning and stinging sharp | 0.9785 | no biopsy | 0.960734 |

| Stressful comment (BART >0.9) | Score | Relaxed comment (BART >0.9) | Score |
|---|---|---|---|
| slightly anxious about results | 0.9785 | no biopsy | 0.960734 |
| patient feels needle | 0.9783 | no biopsy | 0.960734 |
| pinch lot of burning and stinging | 0.9778 | no biopsy | 0.960734 |
| had to take care of dressing on foot and shower. . . .pain is froom foot | 0.9775 | no biopsy | 0.960734 |
| no time to think | 0.9775 | no biopsy | 0.960734 |
| medical doctor consent ( very crowded room ) | 0.9774 | no biopsy | 0.960734 |
| sore | 0.9774 | no biopsy | 0.960734 |
| sore | 0.9774 | no biopsy | 0.960734 |
| sore | 0.9774 | no biopsy | 0.960734 |
| sore | 0.9774 | no biopsy | 0.960734 |
| difficulties to set up | 0.9773 | no biopsy | 0.960734 |
| neck starts hurting | 0.9772 | no biopsy | 0.960734 |
| patient waiting | 0.9771 | no biopsy | 0.960734 |
| patient waiting | 0.9771 | no biopsy | 0.960734 |
| patient waiting | 0.9771 | no biopsy | 0.960734 |
| 2 hours waiting time ! ! ! | 0.9771 | no biopsy | 0.960734 |
| neck is killing me anesthesia | 0.9769 | no biopsy | 0.960734 |
| pinch-burn | 0.9768 | no biopsy | 0.960734 |
| running late | 0.9767 | no biopsy | 0.960734 |
| patient uncomfortable from us pressure | 0.9767 | no biopsy | 0.960734 |
| head-ache | 0.9766 | no biopsy | 0.960734 |
| patient : ouch ! “ | 0.9761 | no biopsy | 0.960734 |
| always feel anxous when at work . why ? | 0.9760 | no biopsy | 0.960734 |
| inner samples were uncomfortable | 0.9760 | no biopsy | 0.960734 |
| patient worried dr won’t be able to place clip | 0.9760 | no biopsy | 0.960734 |
| that hurts | 0.9759 | no biopsy | 0.960734 |
| stomach upset | 0.9755 | no biopsy | 0.960734 |
| worried it is swollen | 0.9751 | no biopsy | 0.960734 |
| have to have it off | 0.9749 | no biopsy | 0.960734 |
| ressure | 0.9749 | no biopsy | 0.960734 |
| exhausted | 0.9744 | no biopsy | 0.960734 |
| exhausted | 0.9744 | no biopsy | 0.960734 |
| pinch burning stining | 0.9743 | no biopsy | 0.960734 |
| long day | 0.9743 | no biopsy | 0.960734 |
| discomfort of the table | 0.9736 | no biopsy | 0.960734 |
| discomfort of the table | 0.9736 | no biopsy | 0.960734 |
| patient crying | 0.9736 | no biopsy | 0.960734 |
| patient crying | 0.9736 | no biopsy | 0.960734 |
| patient crying | 0.9736 | no biopsy | 0.960734 |

| Stressful comment (BART >0.9) | Score | Relaxed comment (BART >0.9) | Score |
|---|---|---|---|
| shaking | 0.9736 | no biopsy | 0.960734 |
| shaking | 0.9736 | no biopsy | 0.960734 |
| shaking | 0.9736 | no biopsy | 0.960734 |
| shaking | 0.9736 | no biopsy | 0.960734 |
| went to bed exhausted after 2 hour drive home | 0.9735 | no biopsy | 0.960734 |
| has sick husband at home | 0.9733 | no biopsy | 0.960734 |
| patient alone again ( headache 2 days ) | 0.9732 | no biopsy | 0.960734 |
| neck and shoulder hurt | 0.9732 | no biopsy | 0.960734 |
| shoulder hurts | 0.9731 | no biopsy | 0.960734 |
| shoulder hurts | 0.9731 | no biopsy | 0.960734 |
| shoulder hurts | 0.9731 | warm | 0.959771 |
| shoulder hurts | 0.9731 | hypnotic induction ( imagery of home in bed ) | 0.959004 |
| painful | 0.9730 | patient feels good | 0.958995 |
| medical doctor : concentrate on going to sleep patient : I ’m not going to sleep ! “ | 0.9730 | felt better | 0.958468 |
| patient very hostile | 0.9728 | under sedatives | 0.957931 |
| patient very hostile | 0.9728 | feeling much better | 0.957798 |
| neck hurts more than procedure | 0.9728 | massage neck | 0.956956 |
| dr. gets angry | 0.9727 | massage neck | 0.956956 |
| sore this evening | 0.9722 | massage neck | 0.956956 |
| i got my period so I ’m very emotional | 0.9722 | feeling better | 0.956622 |
| final exams day | 0.9721 | surg : benign calcs & alh | 0.956618 |
| aggrevation | 0.9719 | no pain | 0.955138 |
| feels punching | 0.9716 | no pain | 0.955138 |
| anxious for results | 0.9715 | no pain | 0.955138 |
| sharp prick | 0.9714 | pleasant day at work | 0.954837 |
| uss right breast | 0.9713 | taking no pain medication | 0.954333 |
| patient not happy about trainee in room | 0.9712 | patient pleased by hypnosis | 0.95369 |
| burning sting sharp | 0.9711 | sounds good | 0.953636 |
| results- I have cancer ! | 0.9706 | laughing | 0.953316 |
| patient is bleeding a lot | 0.9705 | tech very comforting | 0.952959 |
| patient upset that no biopsy taken - does not want removal | 0.9704 | tech very comforting | 0.952959 |
| left rib uncomfortable | 0.9699 | ready for bed | 0.952168 |
| rt . shoulder hurts | 0.9698 | alone | 0.951313 |
| uss | 0.9698 | alone | 0.951313 |
| uss | 0.9698 | alone | 0.951313 |
| uss | 0.9698 | waiting for anesthesia to take effect | 0.950483 |

| Stressful comment (BART >0.9) | Score | Relaxed comment (BART >0.9) | Score |
|---|---|---|---|
| uss | 0.9698 | dr totally ignoring hypnosis | 0.949808 |
| uss | 0.9698 | massages legs | 0.948978 |
| uss | 0.9698 | clean | 0.948904 |
| uss | 0.9698 | had several glasses of wine and brushed my teeth about 30 min ago | 0.948716 |
| uss | 0.9698 | 4 day weekend ! ( labor day ) | 0.948568 |
| uss | 0.9698 | very happy to move arm | 0.948555 |
| uss | 0.9698 | patient feels well | 0.948128 |
| uss | 0.9698 | do not pay attention to the noise here | 0.947428 |
| uss | 0.9698 | benign breast tissue | 0.945237 |
| uss | 0.9698 | just got results : not cancer | 0.94411 |
| uss | 0.9698 | provider left room | 0.943465 |
| salesperson here ( darren ) patient uncomfortable with man in room | 0.9689 | medical doctor enters uss - ¿ thinks it 's benign | 0.943297 |
| neck very bad - ¿ ice pack | 0.9685 | leg massage | 0.942344 |
| neck hurts | 0.9684 | no stinging | 0.942284 |
| neck hurts | 0.9684 | no biopsy necessary | 0.940756 |
| neck hurts | 0.9684 | medical doctor very comforting and encouraging | 0.940659 |
| neck hurts | 0.9684 | probably will not biopsy today | 0.940469 |
| neck hurts | 0.9684 | medical doctor called – reassured that its going as its supposed to | 0.93972 |
| neck hurts | 0.9684 | beginning work day with not so many meetings | 0.938824 |
| neck hurts | 0.9684 | patient unaware that she was here for biopsy | 0.937741 |
| neck hurts | 0.9684 | beach image | 0.937178 |
| neck hurts | 0.9684 | so life is great | 0.936742 |
| neck hurts | 0.9684 | hypnotic induction : beach imagery | 0.936727 |
| neck hurts | 0.9684 | unremarkable breast parenchyma | 0.936554 |
| patient : ow ! : medical doctor : that 's just the local going in | 0.9680 | unremarkable breast parenchyma | 0.936554 |
| tech : well that 's not good | 0.9677 | imagery was warm beach | 0.935859 |
| patient siad she wants to pay attention to what 's going on around . the relaxation exercise makes her feel worse | 0.9676 | just ready to leave work | 0.934901 |
| pulling stinging | 0.9675 | that 's the sharpest part patient : I do not even feel it | 0.933315 |
| like a bee sting ! ( tech ) | 0.9675 | leaves patient alone in room | 0.932707 |
| doxio meds - ¿ not sure if biopsy can be taken - ¿ mds finding out | 0.9674 | cleanin | 0.932637 |
| dr. k : having difficulties | 0.9674 | medical doctor consent : no sharp pain | 0.932599 |

| **Stressful comment (BART >0.9)** | **Score** | **Relaxed comment (BART >0.9)** | **Score** |
|---|---|---|---|
| neck very bad | 0.9673 | got blanket | 0.931995 |
| butterflies in my stomach | 0.9661 | stayed home from work | 0.931395 |
| dog barking too much | 0.9660 | the ice helps | 0.93097 |
| patient told that mds are not sure whther they removed the calcs or not | 0.9659 | tech uses kind of imagery why do not you go to your own personal beach ? you do not have to be here | 0.93002 |
| found out I had cancer . I was in hospital | 0.9658 | looking forward to weekend | 0.929617 |
| difficulties with maschine | 0.9653 | waiting for s.o . to come in | 0.929286 |
| have to go to bank to set up an account for mom . she does not have all the necessary info | 0.9648 | patient happy | 0.928854 |
| patient severe pain | 0.9645 | patient happy | 0.928854 |
| driving in the snow ! | 0.9644 | patient happy | 0.928854 |
| feeling physically exhausted | 0.9639 | medical doctor prep : I promise I wo not hurt you ! | 0.926324 |
| needle stick patient : do not like needles | 0.9635 | feels much better | 0.924134 |
| patient nervous | 0.9628 | getting off the table | 0.923858 |
| patient nervous | 0.9628 | no biopsy – cyst aspiration | 0.918962 |
| patient nervous | 0.9628 | beach imagery | 0.918688 |
| patient nervous | 0.9628 | should not feel sharp | 0.914898 |
| arm hurts | 0.9626 | scans ( no needle yet ) | 0.913452 |
| arm hurts | 0.9626 | more comression | 0.910408 |
| arm hurts | 0.9626 | just leaving a prayer meeting | 0.909721 |
| arm hurts | 0.9626 | shoulder | 0.906583 |
| arm hurts | 0.9626 | not pain | 0.906506 |
| arm hurts | 0.9626 | getting blanket | 0.902996 |
| intolerable | 0.9626 | imagery : beach | 0.90027 |
| feeling pinch | 0.9620 | imagery : beach | 0.90027 |
| racing from one meeting to another | 0.9619 | patient sitting up | 0.900011 |
| wants to talk but no one anwers | 0.9616 | | |
| patient upset that no biopsy taken | 0.9616 | | |
| can not continue | 0.9612 | | |
| i know the results are bad | 0.9608 | | |
| feeling pinchy | 0.9606 | | |
| lots of chores today | 0.9602 | | |
| difficulty breathing ( asthma ) | 0.9601 | | |
| these are cyts . do you really want us to stick you with needles ? | 0.9601 | | |
| my daughter having a bad dream | 0.9599 | | |
| holding pressure | 0.9598 | | |
| holding pressure | 0.9598 | | |
| holding pressure | 0.9598 | | |

| Stressful comment (BART >0.9) | Score | Relaxed comment (BART >0.9) | Score |
|---|---|---|---|
| holding pressure | 0.9598 | | |
| holding pressure | 0.9598 | | |
| holding pressure | 0.9598 | | |
| holding pressure | 0.9598 | | |
| holding pressure | 0.9598 | | |
| holding pressure | 0.9598 | | |
| holding pressure | 0.9598 | | |
| holding pressure | 0.9598 | | |
| holding pressure | 0.9598 | | |
| holding pressure | 0.9598 | | |
| holding pressure | 0.9598 | | |
| holding pressure | 0.9598 | | |
| holding pressure | 0.9598 | | |
| holding pressure | 0.9598 | | |
| holding pressure | 0.9598 | | |
| holding pressure | 0.9598 | | |
| holding pressure | 0.9598 | | |
| most painful is my finger | 0.9596 | | |
| high anxiwty when left hospital…much better now ! | 0.9590 | | |
| patient pressure in legs | 0.9589 | | |
| became anxious at mention of needle | 0.9585 | | |
| wound is now itchy | 0.9582 | | |
| you ’ll feel it going in . uncomfortable for a few seconds | 0.9580 | | |
| neck uncomfortable | 0.9577 | | |
| neck uncomfortable | 0.9577 | | |
| patient afraid of needles | 0.9566 | | |
| head and neck sore | 0.9566 | | |
| patient wish I could move my leg | 0.9562 | | |
| unable to complete script | 0.9561 | | |
| insturctions | 0.9560 | | |
| day 4 and no word from my doctor yet | 0.9557 | | |
| ribs slightly sore - wants to go home | 0.9545 | | |
| holding pressure on first | 0.9545 | | |
| lots of pain | 0.9541 | | |
| getting down to being pissed off at it all | 0.9537 | | |
| 2abscess dr | 0.9533 | | |
| expecting biopsy results today ! | 0.9531 | | |
| sterelize | 0.9526 | | |

| Stressful comment (BART >0.9) | Score | Relaxed comment (BART >0.9) | Score |
|---|---|---|---|
| two lesions – patient to have two biopsies | 0.9524 | | |
| discomfort | 0.9524 | | |
| discomfort | 0.9524 | | |
| discomfort | 0.9524 | | |
| discomfort | 0.9524 | | |
| discomfort | 0.9524 | | |
| discomfort | 0.9524 | | |
| unable to aspirate | 0.9517 | | |
| unable to aspirate | 0.9517 | | |
| did not sleep well | 0.9516 | | |
| first day at work after 10 day vacation | 0.9512 | | |
| difficult to write | 0.9511 | | |
| woman coming to crop need to finish cleaning my house | 0.9504 | | |
| hurts more anesthesia | 0.9501 | | |
| maschine does not work - ¿ tech messed that up | 0.9496 | | |
| just cancelled a vacation because of foot injury – on antibiotics now | 0.9494 | | |
| patient still hostile | 0.9494 | | |
| patient says that she ca not concentrate on the exercise she wants to apy attention to what 's going on in the room . unable to finish script | 0.9492 | | |
| sore neck due to positioning | 0.9491 | | |
| thinking about my ovarian cancer test | 0.9488 | | |
| medical doctor : sharp ? patient : that was prickly | 0.9486 | | |
| sharp pain | 0.9486 | | |
| medical doctor not comfortable proceeding with biopsy . procedure finished | 0.9484 | | |
| asthma is starting | 0.9481 | | |
| patient coughing | 0.9471 | | |
| headache | 0.9468 | | |
| headache | 0.9468 | | |
| headache | 0.9468 | | |
| headache | 0.9468 | | |
| arm uncomfortable | 0.9459 | | |
| pressing hand | 0.9446 | | |

| Stressful comment (BART >0.9) | Score | Relaxed comment (BART >0.9) | Score |
|---|---|---|---|
| suffering from severe seasonal allergies | 0.9445 | | |
| suffering from severe seasonal allergies | 0.9445 | | |
| had to reallign | 0.9442 | | |
| took valium prior to procedure for anxiety | 0.9441 | | |
| neck terrible | 0.9436 | | |
| under compression | 0.9435 | | |
| feels wired | 0.9432 | | |
| unable to biopsy | 0.9429 | | |
| unable to biopsy | 0.9429 | | |
| unable to biopsy | 0.9429 | | |
| unable to biopsy | 0.9429 | | |
| wants to get it over | 0.9425 | | |
| difficulty repositioning | 0.9425 | | |
| headache this morning | 0.9421 | | |
| lots of traffic on way home | 0.9419 | | |
| neck and shoulders sore | 0.9413 | | |
| long day-evening class | 0.9411 | | |
| with no results yet I have small worry in the back of my mind ... | 0.9405 | | |
| patient does not want to be asked anymore | 0.9402 | | |
| arm in pain | 0.9394 | | |
| results negative ! | 0.9392 | | |
| off scale | 0.9391 | | |
| very tired | 0.9386 | | |
| very tired | 0.9386 | | |
| very tired | 0.9386 | | |
| gravity is making my breast sag a bit | 0.9384 | | |
| bleeding ++ with biopsy | 0.9367 | | |
| patient felt needle | 0.9366 | | |
| upset will not get results this week – probably not until first week in november | 0.9363 | | |
| back hurts | 0.9361 | | |
| back hurts | 0.9361 | | |
| back hurts | 0.9361 | | |
| back hurts | 0.9361 | | |
| awake since 3am – bad dreams | 0.9357 | | |
| trying to find position | 0.9342 | | |
| gh discomfort | 0.9338 | | |
| tech angry | 0.9335 | | |

| Stressful comment (BART >0.9) | Score | Relaxed comment (BART >0.9) | Score |
|---|---|---|---|
| needs a lot of attention | 0.9331 | | |
| crying because of pain | 0.9330 | | |
| can you give me a reason why my anxiety should have changed ? patient very hostile | 0.9326 | | |
| patient discomfort due to positioning | 0.9324 | | |
| dr needs help from dr | 0.9322 | | |
| two more days for results – does clip mean radiologist knew from xrays more needs to be done or ? ! ? ! | 0.9321 | | |
| mother was home and had to talk about it | 0.9316 | | |
| test at school | 0.9316 | | |
| yankees losing to marlins | 0.9315 | | |
| dr k too many vessels | 0.9309 | | |
| rushing to tidy house for cleaning help | 0.9307 | | |
| shivering | 0.9301 | | |
| try not to think about the test | 0.9300 | | |
| needle for biopsy does not open and has to be replaced | 0.9298 | | |
| neck hurts massaging | 0.9297 | | |
| tech : does that sting ? “ | 0.9297 | | |
| right shoulder hurts | 0.9295 | | |
| itchy | 0.9291 | | |
| itchy | 0.9291 | | |
| itchy | 0.9291 | | |
| patient complains about compression | 0.9287 | | |
| patient wanted biopsy to be over | 0.9265 | | |
| itching . pulled off over bandage . saw breast . thinking . praying | 0.9259 | | |
| nobody listens | 0.9254 | | |
| waiting for 2nd biopsy | 0.9252 | | |
| neck very sore | 0.9252 | | |
| afraid of being hurt by child I am ta for | 0.9245 | | |
| 2nd biopsy not possible | 0.9234 | | |
| burning sensation | 0.9226 | | |
| burn | 0.9213 | | |
| burn | 0.9210 | | |
| very disappointed | 0.9201 | | |
| pain | 0.9201 | | |

| Stressful comment (BART >0.9) | Score | Relaxed comment (BART >0.9) | Score |
|---|---|---|---|
| pain | 0.9201 | | |
| shoulders hurt | 0.9199 | | |
| pain during biopsy | 0.9194 | | |
| very sore | 0.9181 | | |
| child sick | 0.9174 | | |
| had pain | 0.9172 | | |
| hurt and pain | 0.9165 | | |
| procedure cancelled – not possible to biopsy | 0.9163 | | |
| does not want to be videotaped – double biopsy | 0.9157 | | |
| burning sensation patient : it hurts | 0.9154 | | |
| results – maligt | 0.9152 | | |
| results – maligt | 0.9152 | | |
| hot | 0.9142 | | |
| patient half an hour late | 0.9120 | | |
| pain | 0.9117 | | |
| pain | 0.9117 | | |
| felt some burning on 3rd | 0.9117 | | |
| patient neck | 0.9109 | | |
| sharp pai during incision | 0.9108 | | |
| very tired and weak feeling | 0.9107 | | |
| ribs hurt | 0.9107 | | |
| only hurts when I move a lot | 0.9103 | | |
| major surgery earlier this year | 0.9100 | | |
| patient does not like position | 0.9095 | | |
| have not heard yet ! busy getting ready for a large party at our home | 0.9085 | | |
| hot flash | 0.9080 | | |
| waiting to get into the bathroom | 0.9080 | | |
| severely atypical intraductal proliferation bordering on ductal carcinoma in-situ | 0.9076 | | |
| dict ectasia | 0.9051 | | |
| just got home from town meeting – took a slide in wet leaves scraping leg | 0.9042 | | |
| patient difficulty with imaging | 0.9041 | | |
| still no word from the doctor | 0.9041 | | |
| dr interrupts again | 0.9025 | | |
| sore breast | 0.9024 | | |
| sore breast | 0.9024 | | |
| tired | 0.9022 | | |
| tired | 0.9022 | | |

| **Stressful comment (BART >0.9)** | **Score** | **Relaxed comment (BART >0.9)** | **Score** |
|---|---|---|---|
| tired | 0.9022 | | |
| tired | 0.9022 | | |
| tired | 0.9022 | | |
| tired | 0.9022 | | |
| tired | 0.9022 | | |
| long compression | 0.9018 | | |
| patient asks for dr 1 does not want dr 2 to do the procedure | 0.9014 | | |
| pain at biopsy site | 0.9009 | | |
| needle in pain | 0.9008 | | |

# 2 SUPPLEMENTARY TABLES AND FIGURES

## 2.1 Tables

Table S1. Data collected along the CNB pathway.

| Type of data | Data collected |
|---|---|
| Socio demographic baseline | Sex, Age, Weight, Ethnic / Race, Marital status, Menopause status, Hormone replace therapy (HRT), Oral contraceptive pills (OCP), Menarche age, Alcohol, Smoking, Hours of sleep Clinical experience: first procedure or repeat, diagnosis with ultrasound or mamography |
| Pre-procedure | Psychological instruments: Impact of Event Scale distress that is caused by traumatic events (IES), Perceived Stress Scale a classic stress assessment instrument (PSS), STAI- State-Trait Anxiety Inventory, and Center for Epidemiological Studies-Depression Scale (CESD). Patient-report: Anxiety and Pain verbal self-rate on a scale from 0 to 10. Physiological: Blood pressure systolic and diastolic, heart rate. Biochemical: Salivary cortisol. Textual comments observations pre-procedure, phrases mentioned by patients and by clinicians (unstructured data). |
| During procedure | Patient-report: Anxiety and pain verbal self-rate collected every 10 min on a scale from 0 to 10, where rating of 0 indicated “no pain” or “no anxiety” and 10 indicated “worst possible pain,” or “worst possible anxiety”. Textual observations: patient comments noted by technical radiologist, medication use for sedation and analgesics. Clinical process: time point of local anesthesia and core biopsy, total procedure time, interactions with the medical team (Provider, MD, Fellow or Technician) in terms of negative suggestions from the medical team, negative “adherence”, positive suggestions, encouragement, and praise. Where praise are comments that label a person (“you are a great patient)” rather than mentioning an action a person does (encouragement - “thanks for holding still”). |
| Post-procedure | Patient-report: Anxiety and Pain verbal self-rate of comfort level on a scale from 0 to 10. Physiological: Blood pressure systolic and diastolic, heart rate. Biochemical: Salivary cortisol. Textual observations at the end of the procedure, some phrases mentioned by patients and by clinicians (unstructured data). |
| At home | Patient-report: Anxiety and pain verbal self-rate of comfort level on a scale from 0 to 10. Biochemical: Salivary cortisol 3 times per day. Textual observations from patients written in a diary card (unstructured data). |

Table S2: **Participant-specific view of the model application** for the patient with the maximum association value for **a.** “stressful” and **b.** “painful”.

| **Patient** | **Comment registered** | **Moment in the procedure** | **Semi-automated classification** |
|---|---|---|---|
| **a. Patient 1135 with maximum association value for “stressful”** | Back pain | **Pre-biopsy phase** | Painful |
| | Back hurts | **Biopsy Phase** Min. 10 | Painful, Stressful |
| | Difficult repositioning | Min. 20 | Stressful |
| | Patient worried about surgery | Min. 40 | Stressful |
| | Back hurts, sting, burn | | Painful, Stressful |
| | Patient wants lots of local anesthesia | | BART values below threshold |
| | Anesthesia, patient asks for lots of meds | Min. 60 | BART values below threshold |
| | It’s a training session, patient worried Dr H won’t be able to place clip, angry that Dr B isn’t doing it | Min. 70 | BART values below threshold, Stressful, Painful |
| | Band-aid, crying because of pain, laughing at same time, shaking | **Post-biopsy Phase** Recovery | Painless, Painful, Stressful, BART values below threshold |
| Observer reported only | Patient very picky, has sick husband at home, needs a lot of attention, patient very pleased with hypnosis | General Observations | BART values below threshold, Stressful |
| Patient self-reported in the diary | Work-related stress | **At Home Phase** Day 2 8 am | Stressful |
| | About to give a reception for 55 people: stress | Day 3 8 am | Stressful |
| | Reception over but still reved up | Day 3 11 am | BART values below threshold |

| Patient | Comment registered | Moment in the procedure | Semi-automated classification |
|---|---|---|---|
| | May get biopsy results in afternoon | Day 4 8 am | BART values below threshold |
| | May hear soon | Day 4 11 am | BART values below threshold |
| | No cancer! | Day 4 3 pm | BART values below threshold |
| | I feel great | Day 4 9 pm | Relaxed |
| | The world has a wonderful shimmer to it | Day 5 8 am | BART values below threshold |
| | Off to an art gallery | Day 5 11 am | BART values below threshold |
| | Rested and relaxed | Day 5 3 pm | Relaxed |
| **b. Patient 1134 with maximum association value for "stressful"** | No biopsy, patient does not know anything about procedure, anxious and angry none informed her in a better way | **Pre-biopsy phase** | Painless, Relaxed, BART values below threshold, Stressful |
| | Tech very empathic, patient problems with neck | **Biopsy Phase** Min. 10 | BART values below threshold |
| | Patient complains about arm falling asleep, providers out of room, Dr. K tells patient they probably can't do biopsy | Min. 20 | BART values below threshold, Relaxed |
| | Dr. K and Dr. H enter | Min. 30 | BART values below threshold |
| | Provider looking at pictures, got them, can take biopsy | Min. 40 | BART values below threshold |
| | Setting up machine, anesthesia, incision | | BART values below threshold |
| | Neck very bad $\rightarrow$ ice pack, no biopsy | Min. 60 | Painful, Stressful, Painless, Relaxed |
| | Band-aid | **Post-biopsy Phase** Recovery | Painless |
| Observer reported only | No biopsy taken | General Observations | Relaxed |

| **Patient** | **Comment registered** | **Moment in the procedure** | **Semi-automated classification** |
|---|---|---|---|
| Patient self-reported in the diary | Feeling very tired | **At Home Phase** Day 1 3 pm | BART values below threshold |
| | Took a nap, feel better | Day 1 9 pm | Relaxed |
| | Slept well | Day 2 8 am | Relaxed |
| | Just booked my surgery | Day 3 3 pm | BART values below threshold |
| | All of a sudden I am getting pain when the needle was injected first time such pain | Day 4 8 am | Painful, Stressful |

## 2.2 Figures

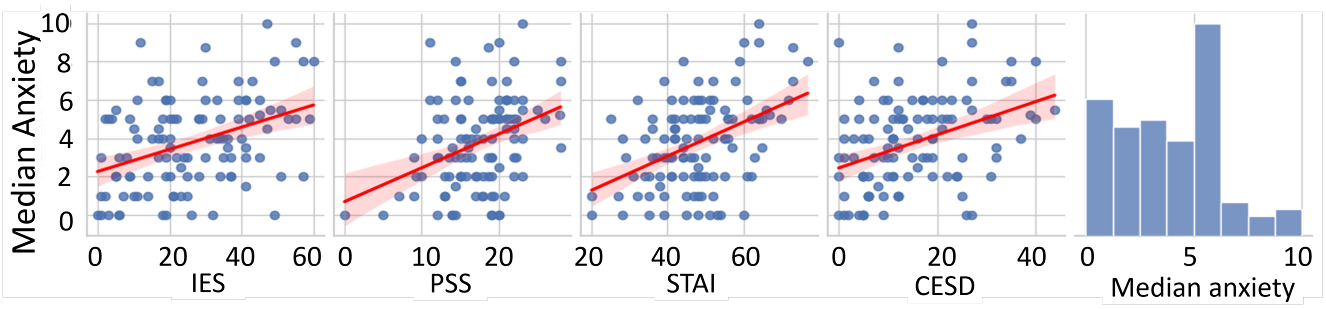


Figure S1: **Correlation between psychological pre-assessments and median anxiety.** Each psychological construct was analyzed independently as a significant predictor for anxiety, resulting in four distinct models for State-Trait Anxiety Inventory (STAI), Impact of Event Scale (IES), Center for Epidemiologic Studies Depression Scale (CES-D), and Perceived Stress Scale (PSS).

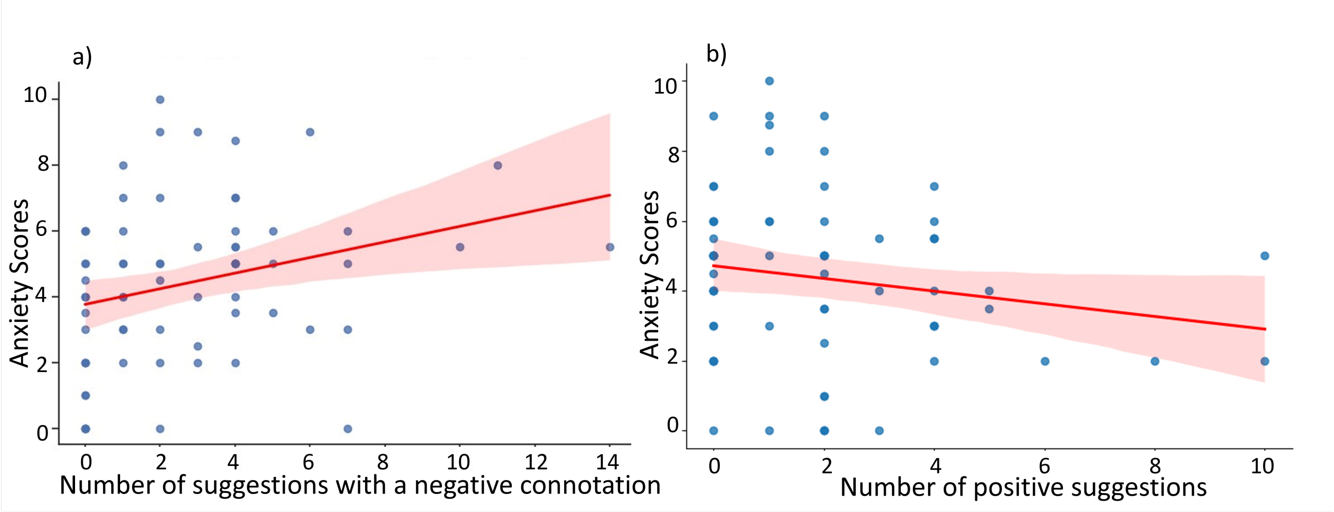


Figure S2: **Healthcare professionals choice of words and median anxiety.** Graph **(A)** depicts maximum counted negative suggestions (e.g., "this will hurt") mentioned by healthcare professionals during CNB and corresponding median anxiety, while **(B)** depicts counted positive suggestions (e.g., "you may feel a prick" and corresponding median anxiety per patient.

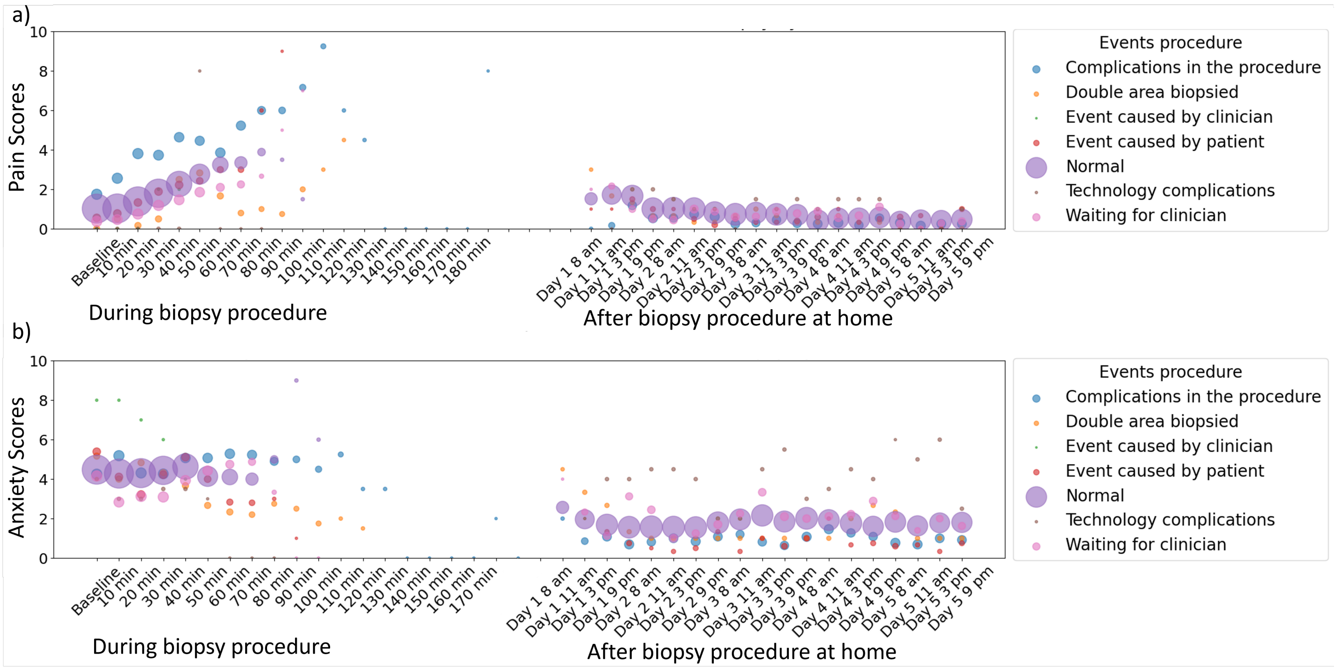


Figure S3: **Sub-classification per critical event registered during CNB.** Figure **(A)** graphs pain and figure **(B)** graphs anxiety for normal biopsy procedures sub-categorized by events during the procedure. Sizes of the bubbles represent population size per moment, events were sub-categorized through semi-automated unstructured data analysis.

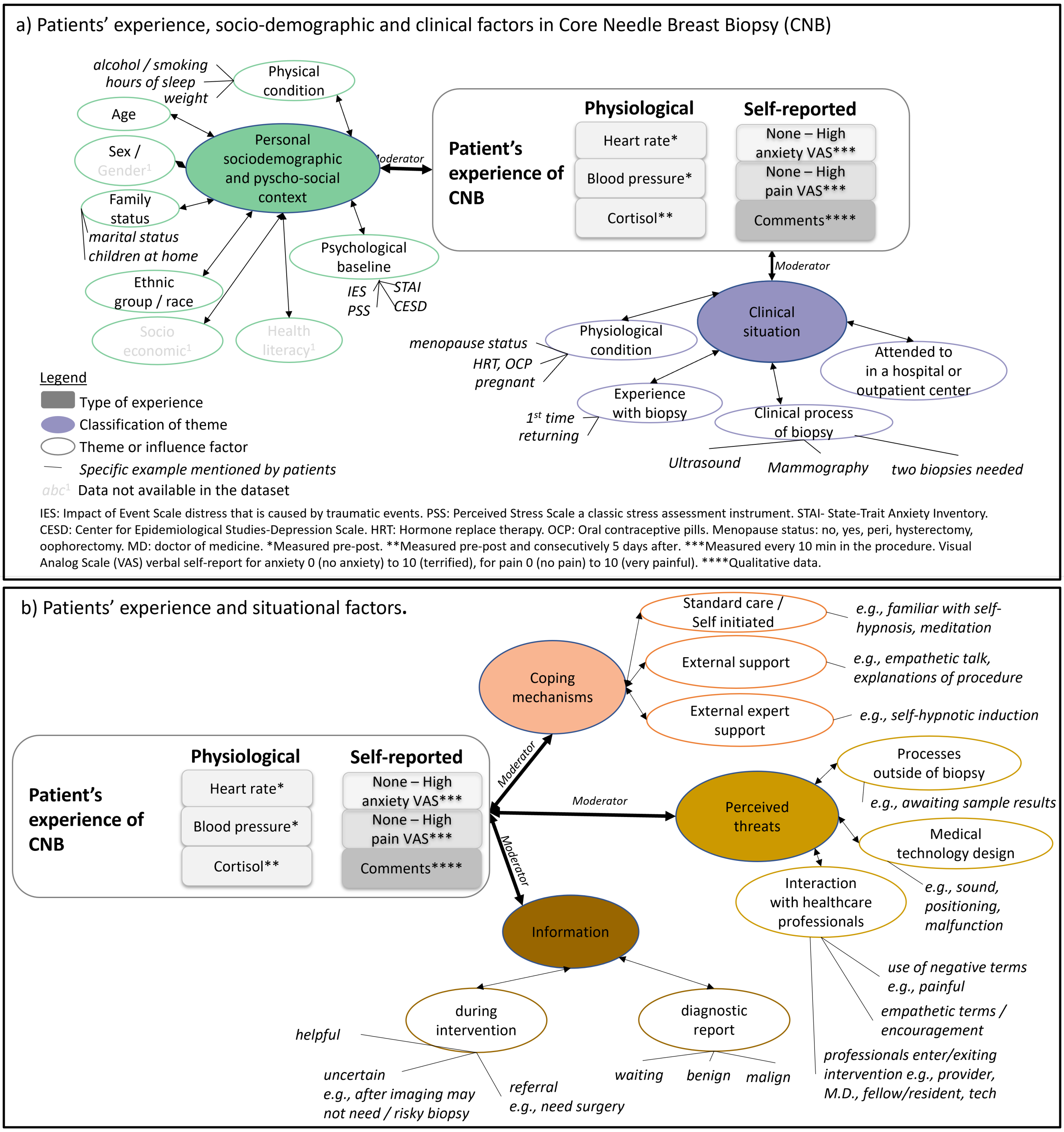


Figure S4: **Factors moderating patients' experience in CNB.** Diagram **(A)** maps patients' experience and factors moderating the experience: sociodemographic, psychosocial, and clinical, while **(B)** maps situational factors present during the procedure (e.g., coping strategies, perceived threats, and information).

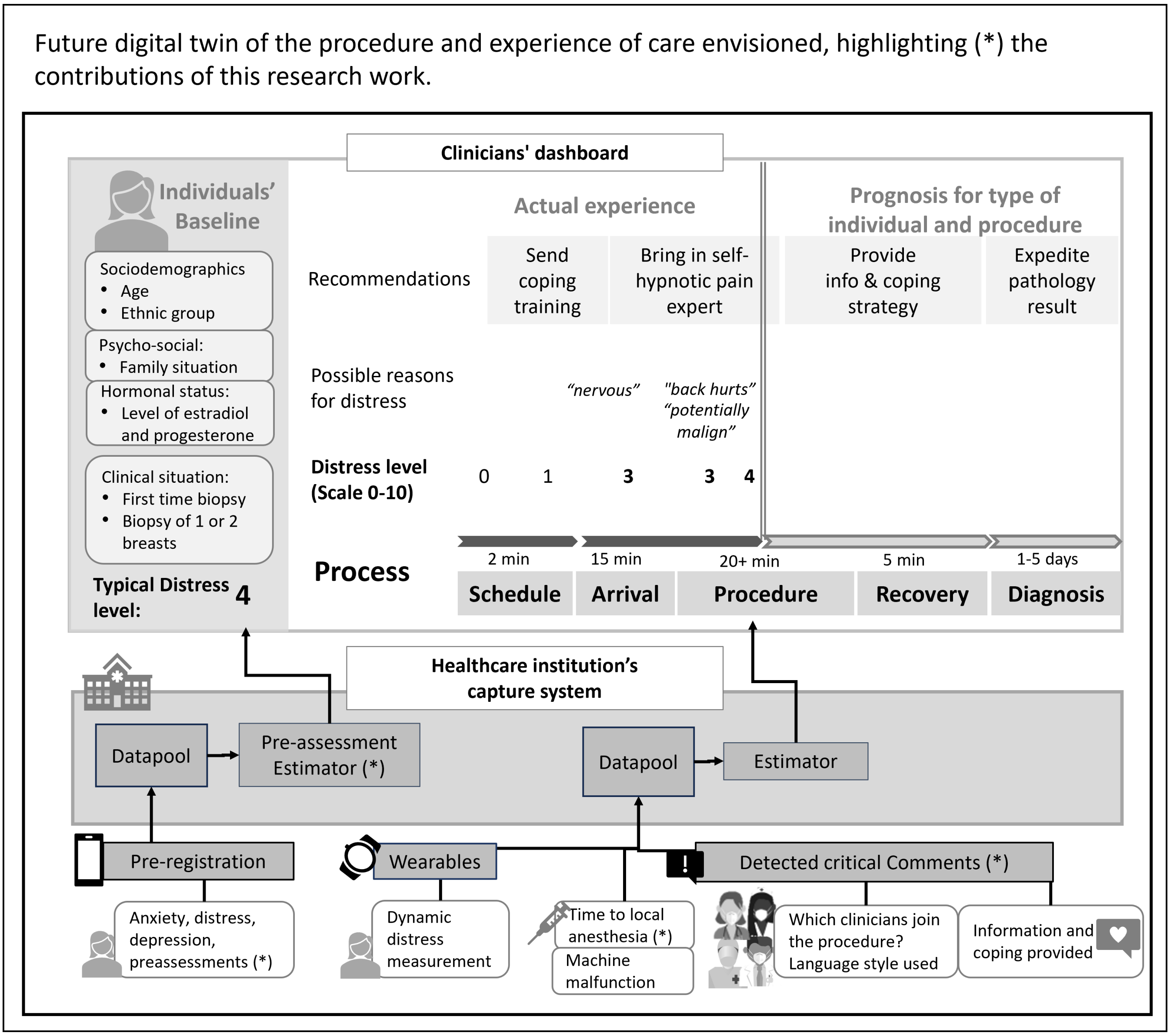


Figure S5: **Envisioned model of pathway and experience**. Digital simulations and modeling of medical procedures like the one explored in this paper, and envisioned this figure may transform established ways of operating healthcare services by providing transparency, clarity in the relations of patient-provider, or predictions of events.
